# Structure–Performance Relationships of Metal Oxide Nanofluids for Enhanced Oil Recovery: Balancing Colloidal Stability, Interfacial Regulation, and Pore Transport

**DOI:** 10.3390/ma19194199

**Published:** 2026-09-30

**Authors:** Caiping Hu, Bo Zhou, Minghui Lv, Liping Zeng, Pindong Tan, Feng Zheng, Shuai Gao, Yu Sun, Jinjian Hou, Shuo Liu, Xiaobin Li

**Affiliations:** 1 Shandong Provincial Geo-Mineral Engineering Exploration Institute, Shandong Provincial Bureau of Geology & Mineral Resources, Jinan 250014, China; 2 Shandong Engineering Research Center for Environmental Protection and Remediation on Groundwater, Jinan 250014, China; 3 Key Laboratory of Geological Disaster Risk Prevention and Control, Emergency Management Department of Shandong Province, Jinan 250014, China; 4 School of Chemistry and Chemical Engineering, Hainan University, Haikou 570228, China; 5 School of Chemical Engineering, Tianjin University, Tianjin 300072, China; 6 Jining District Inspection and Testing Center, Ulanqab 012000, China; 7 School of Business, East China University of Science and Technology, Shanghai 200030, China

**Keywords:** metal oxides, nanofluids, enhanced oil extraction, colloid stability, interface regulation, migration of porous media, oil and water separation

## Abstract

**Highlights:**

- Four metal oxide nanofluids were compared under identical experimental conditions.
- SiO_2_ balanced colloidal stability, interfacial regulation, and deep pore transport.
- At 0.30 wt%, SiO_2_ achieved 52.28% oil recovery versus 18.35% for water flooding.
- Moderate adsorption favored deep propagation more than maximum adsorption capacity.
- SiO_2_ retained over 90% dispersion stability after 30 days of aging.

**Abstract:**

The effect of nanofluid assistance in improving the recovery rate depends on the balance between colloidal stability, interfacial regulation, and the transport capacity of nanoparticles in porous media. Under the same preparation and experimental conditions, this study systematically compared four nanofluids of SiO_2_, TiO_2_, Al_2_O_3_, and Fe_3_O_4_ to establish their structure-performance relationship. Through a variety of complementary characterization methods and core flooding experiments, the colloidal properties, rheological behavior, interfacial characteristics, adsorption characteristics, transport ability, and oil displacement effect of each system were evaluated. The results show that the comprehensive performance of SiO_2_ nanofluids is the best, which is closely related to its good dispersion stability, interfacial regulation ability, and effective propagation in porous media. At a concentration of 0.30 wt%, the final recovery rates of SiO_2_, TiO_2_, Al_2_O_3_, and Fe_3_O_4_ were 52.28%, 41.01%, 31.54%, and 21.49%, respectively, while the water flooding recovery rate was only 18.35%. Although Fe_3_O_4_ shows strong adsorption and retention ability, particle aggregation and pore-throat retention limit its effective transport; in contrast, the moderate adsorption of SiO_2_ not only maintains continuous interfacial regulation, but also avoids excessive retention. After 30 days of aging, the dispersion stability of SiO_2_ remains above 90%. The above results show that the nanoparticle screening used for EOR should comprehensively consider the synergistic effect among colloidal stability, interfacial activity, adsorption-retention behavior, and deep pore transport, and should not rely on a single performance parameter.

## 1. Introduction

With the continuous improvement of oilfield development, many conventional reservoirs have entered the high water content period [1]. Primary oil extraction mainly relies on the natural energy of the reservoir, while secondary water driving maintains the stratum pressure and replaces crude oil through external water injection. However, the inhomogeneity of the reservoir and the complex structure of the pores and throats will make the injected water move first along the high-permeability channel, resulting in premature water detection, ineffective circulation, and reduced spillover efficiency. Fu et al. pointed out that the priority circulation channel will gradually develop during the water-driving process, which will significantly affect the use of residual oil in the inhomogeneous reservoir [2]. Similarly, long-term water-driving experiments show that the priority circulation channel and runaway will reduce the spillage efficiency, causing a large amount of residual oil to remain in the inhomogeneous porous structure [3]. Therefore, a considerable part of crude oil after water drive may remain in the pores in the form of oil film, isolated oil droplets, corner oil, and blind end retention oil. This stability depends not only on capillary pressure but also on the oil wettability of the rock surface, adsorption of heavy components, and the strength of the oil–water interface film [4]. Polymer flooding, surfactant flooding, and alkaline composite flooding can respectively improve fluidity ratio, reduce interfacial tension, or alter interfacial reactions, but high temperature, high mineralization, and complex mineral surfaces can cause polymer degradation, surfactant adsorption, and reduced formulation compatibility [5]. Some systems may also cause excessive emulsification and increased injection pressure. Therefore, how to reduce crude oil interface constraints, expand the low-permeability zone coverage while ensuring injectable quality, and suppress the dominant channel is the core issue that must be addressed to improve recovery after water flooding.

Recent research has explored formulation systems containing nanoparticles through different EOR strategies. Wang et al. constructed a composite system composed of amphoteric copolymer nanospheres and betaine surfactants to improve oil recovery [6]. Ramadhani et al. proposed a nano-intelligent water system containing amphoteric surfactants and pointed out the importance of regulating fluid–fluid and rock–fluid interactions [7]. Shafaay et al. further studied a nanoparticle–surfactant–biopolymer composite system to improve oil recovery [8]. These studies show that nanoparticles can participate in a variety of interfacial and transport processes, not just as traditional viscosity regulators. Nanoparticles have large specific surface areas and high surface energy. The hydroxyl groups, metal active sites, and charge layers on the particle surface can participate in the rearrangement of the oil–water–rock three-phase interface, enabling them to simultaneously have potential functions such as wettability regulation, interfacial tension reduction, oil film stripping, structural separation pressure formation, and fluid viscosity reduction. Compared to conventional chemical oil displacement agents, the amount required for nanoparticles is usually lower and can be adjusted through particle size, crystal shape, and surface chemistry. However, nanofluids are not uniformly homogeneous liquids with fixed properties. Inter-particle van der Waals interactions, magnetic interactions, and electrostatic repulsion determine their initial dispersion state [9,10]. pH, salt type, ion intensity, temperature, and shear history all alter the thickness of the electric double layer, the structure of the hydration layer, and the rate of particle aggregation. After particles enter the reservoir, they undergo convective migration, Brownian diffusion, pore wall adsorption, mechanical capture, and re-release [11]. Thus, higher surfactancy does not necessarily translate into higher core recovery: when adsorption is too weak, particles struggle to continuously alter the rock surface; when adsorption is too strong, particles may remain near the well or at the throat entrance, losing the ability to spread into the deep remaining oil zone. What truly determines the effectiveness of oil displacement is whether an appropriate balance is formed among dispersion stability, interfacial action, rheological regulation, and pore migration [12].

Focusing on nanofluidic enhanced oil production, previous studies have focused on the wetting regulation of silica particles and core displacement behavior. Youssif et al. used porous SiO_2_ nanofluids to replace sandstone, achieving a high additional recovery rate near 0.1 wt%, attributing this to the regulation of fluid viscosity and rock surface state by nanoparticles [13]. Azarshin et al. functionalized SiO_2_ with an amino group and applied it to oily wet carbonate rocks. The results showed that surface modification significantly enhanced the interaction between particles and mineral surfaces and improved crude oil utilization after water flooding [14]. These studies confirm the potential of SiO_2_ in wetting reversal and interface regulation, and also indicate that particle surface chemistry has a greater impact on final oil displacement than simply reducing the primary particle size. However, functionalized particles, brine conditions, and lithology often couple with each other, making strict horizontal comparisons between different materials difficult [15].

The rheological behavior of nanofluids is also significantly affected by the structure and surface properties of nanoparticles. Akande et al. prepared water-based nanofluids from wheat straw charcoal, rice husk charcoal, and commercial activated carbon as raw materials, and systematically investigated the effects of particle concentration, pH, particle characteristics, and shear conditions on viscosity [16,17,18]. The results show that viscosity enhancement depends not only on particle concentration, but also on particle porosity, surface chemical properties, particle size, and particle-fluid interaction. Among them, the activated carbon nanofluid shows a significant viscosity-enhancing effect, which is mainly due to the highly porous structure of its particles. The above findings show that the rheological behavior of nanofluids cannot be explained only from the perspective of particle concentration, but also needs to consider the physicochemical properties of dispersed particles and their interaction with the continuous phase. However, improving the viscosity alone does not necessarily improve the oil displacement performance, because excessive flow resistance or particle retention may both damage the injection capacity and the deep transport in the porous medium. Therefore, rheological regulation should be evaluated in combination with particle transport and displacement performance.

The electrolyte environment is another important factor in controlling the properties of nanofluids [19,20]. The viscosity behavior of ZrO_2_ and γ-Al_2_O_3_ nanoparticles in different electrolyte solutions proved that that the type of nanoparticles and electrolyte composition jointly affect the rheological response. The results show that the influence of electrolytes cannot be generalized between different oxide nanoparticles, because the interaction between the particle surface, ions, and the surrounding liquid phase is material-dependent. This understanding is particularly relevant to the application of reservoirs, because changes in ion composition may change the interparticle interaction, colloidal stability, and rheological behavior. However, there is still a lack of full understanding of the subsequent relationship between the colloidal changes caused by electrolytes and the transport, retention, wettability regulation, and oil displacement of nanoparticles in porous media.

In addition to reservoir displacement, nanomaterials also regulate the oil–water interface for oil–water treatment, including oil-bearing wastewater and produced fluids. Sodium dodecylbenzenesulfonate could form colloidal bubbles, and the effects of initial oil concentration, solution pH, salinity, temperature, and ultrasound on oil removal rate, and used graphene adsorption of oil in the foam phase to achieve regeneration and reuse of surfactant solutions. This study shows that oil droplet size, surface charge, interfacial tension, and mass transfer conditions jointly determine oil–water separation efficiency. Ultrasound can improve subsequent flotation performance by altering the dispersion state of oil droplets. Although the operation of colloidal bubble systems differs from that of nano-oil displacement systems, they share several fundamental processes, including interfacial adsorption, oil droplet collisions, double-layer adjustment, and dispersion stabilization [21,22,23]. Therefore, if only the mining effect of nanofluids in the core is evaluated, while ignoring their impact on oil droplet behavior and reusability in the extracted fluid, the judgment of the actual applicable range of materials remains incomplete [24].

The above research has promoted the development of nanofluids from various aspects such as wettability, viscosity, electrolyte response, and oil–water separation, but consistent material evaluation standards have not yet been formed across these results. First, particle types, primary particle sizes, base liquid composition, and core conditions vary greatly among different studies, and the performance rankings of a single material in different literature are not comparable. Second, static contact angles, interfacial tension, or adsorption amounts are often directly used to explain changes in recovery rates, while adsorption losses, mechanical retention, pressure response, and deep propagation under dynamic flow conditions are not fully accounted for. Third, stronger adsorption is usually interpreted as facilitating wetting reversal, but it may simultaneously cause proximal retention and reduced seepage capacity; higher viscosity helps improve the flow ratio and may also increase injection pressure. Improvements in a single performance may be accompanied by the loss of another. Finally, high temperature, high salinity, aging, and recycling are often studied in a scattered manner, lacking correspondence between particle surface charge changes and macroscopic degradation in displacement performance. What existing research truly lacks is not more isolated performance data, but rather identifying the constraint relationship among “surfactance—adsorption retention—deep migration—harvest effect” within the same experimental boundary [25,26,27,28,29].

Studies have shown that the effect of nanoparticle-assisted EOR is closely related to particle type, surface properties, particle size, and operating conditions. Kandiel et al. have systematically reviewed the application of nanoparticles in EOR, pointing out that the differences in the physicochemical properties of nanoparticles and the different reservoir conditions will significantly affect colloidal stability, wettability transformation, interfacial behavior, and ultimately change the crude oil recovery rate [30]. Bayat et al. conducted a direct comparison of SiO_2_, TiO_2_, and Al_2_O_3_ nanofluids in limestone under the same experimental conditions, and found that the three materials showed obvious differences in particle adsorption, wettability change, interfacial tension, and recovery rate [31,32,33,34,35]. In addition, Kazemzadeh et al. compared Fe_3_O_4_-based and SiO_2_-based nanofluids and proved that the differences in the physicochemical properties of the two types of particles can trigger different EOR mechanisms [36,37,38,39].

Based on the above background, this study selects SiO_2_, TiO_2_, Al_2_O_3_, and Fe_3_O_4_ as representative metal oxide nanoparticles for comparative evaluation. Among them, the surface of SiO_2_ is rich in silanol groups, and SiO_2_ is a hydrophilic oxide and has a relatively clear role in wettability and interfacial regulation; TiO_2_ and Al_2_O_3_ show different surface acid-base responses and charge characteristics, which will directly affect colloidal stability and the interaction between particles and mineral surfaces. The introduction of Fe_3_O_4_ further enriches the contrast dimension. Its magnetic characteristics bring interactions between particles that are different from those of non-magnetic oxides, which may affect the aggregation, retention, and transport of particles in porous media. Therefore, these four materials together form a set of controlled comparison systems with differences in surface chemistry, colloidal behavior, and transport characteristics, which are suitable for EOR-related mechanism analysis. All nanofluids are compared using the same base fluid, preparation process, and experimental conditions. The study does not use a single highest indicator as the material selection criterion, but instead analyzes performance differences along the continuous chain of particle structure, surface chemistry, colloid stability, rheological behavior, interfacial properties, adsorption kinetics, pore migration, and core displacement. X-ray diffraction and infrared spectroscopy are used to identify crystal types and surface functional groups, while hydrodynamic particle size, ζ potential, and polydispersity index are used to characterize the dispersion state of the particles; viscosity, friction factor, contact angle, and interfacial tension are used to evaluate flow and interface regulation; static and dynamic adsorption, permeability, residual resistance, and pressure evolution are used to track the actual behavior of particles after entering the porous medium. On this basis, further investigation is conducted on the effects of temperature, salinity, pH, confining pressure, injection rate, plug size, core permeability, aging time, and recycling, with oil–water separation evaluated as an extended evaluation of material interface interaction capacity. The key question this paper aims to answer is: why particles with strong adsorption capacity may not necessarily achieve higher recovery rates, and which particles can achieve a more reasonable match between surfactant, flow regulation, and deep propagation. This comparative framework helps advance nanofluidic research from single-performance screening to material selection based on structure-activity relationships and pore migration.

## 2. Materials and Methods

### 2.1. Materials and Nanofluid Preparation

A schematic illustration of the main experimental systems used in this study is provided in Figure S1 of the Supporting Information to facilitate understanding of the experimental workflow and setup configuration.

Four metal oxide nanoparticles, SiO_2_, TiO_2_, Al_2_O_3_, and Fe_3_O_4_, were used in this study. The nominal primary particle sizes of SiO_2_, TiO_2_, Al_2_O_3_, and Fe_3_O_4_ nanoparticles provided by suppliers are 20, 25, 30, and 20 nm, respectively. Choosing a narrower primary particle size range (20–30 nm) is intended to minimize the impact of primary particle size when comparing the intrinsic effect of oxide composition and surface chemistry. However, because nanoparticles may form hydration or aggregation structures after dispersion in the aqueous phase, the nominal primary particle size is not considered to be an effective particle size to control the movement in the porous medium, with nominal purity not less than 99%.

Crude oil is taken from the Dagang Oilfield in Tianjin, China. The experiment uses a natural sandstone core, with a diameter of 25.0 ± 0.1 mm and a length of 50.0 ± 0.5 mm. Unless otherwise stated, the gas permeability of the reference core is controlled at 500 ± 25 mD. For detailed information on chemical reagents, crude oil pretreatment, and rock core cleaning, please refer to Section S1 and Table S1 of Supplementary Materials.

Before preparation, the nanoparticles are vacuum-dried at 105 °C for 12 h, and then dispersed in deionized water or the required electrolyte solution. The dispersion is first stirred magnetically at 1000 rpm for 30 min, and then processed with a probe-type ultrasonic processor. The ultrasonic conditions are 20 kHz, 500 W, amplitude 40%, pulse mode 5 s on/3 s off, lasting 60 min. During the ultrasound process, an ice water bath is used to maintain the temperature at 25 ± 2 °C.

The concentration of nanoparticles is treated as an independent formulation parameter because it also affects the colloidal interactions, interfacial activity, rheological behavior, and the retention of particles in porous media. The concentration-dependent experiment was carried out in the range of 0.01–0.50 wt%, and the representative concentration point was selected according to the measured properties. In order to directly compare SiO_2_, TiO_2_, Al_2_O_3_, and Fe_3_O_4_, 0.30 wt% is uniformly used as the reference concentration. The benchmark concentration does not represent the optimal concentration of each material, and the concentration effect has been evaluated separately within the scope of the study. Unless otherwise stated, the pH of the reference nanofluid is adjusted to 8.0 ± 0.1.

In the particle size effect experiment, the samples are grouped according to the peak hydrodynamic particle size measured by dynamic light scattering (DLS), rather than according to the nominal primary particle size provided by the supplier. This treatment is because the effective particle size in the aqueous phase is affected by hydration and aggregation, so it can directly reflect the accessibility and behavior of the particles in the porous medium. By changing the ultrasonic energy input and graded centrifugation conditions, hydrodynamic particle size grouping of about 50–1000 nm was obtained. Before use, re-measure the peak hydrodynamic particle size and polydispersity index, and only select samples with a particle size deviation within 10% of the target value. For detailed salinity gradient, pH adjustment steps, concentration point, and particle size preparation method, please refer to Section S1 of the Supplementary Material.

The Supplementary Material Figure S1 gives a diagram of the main experimental system, covering the preparation and characterization of nanofluids, core displacement, and oil–water separation.

### 2.2. Physicochemical Characterization of Nanofluids

The structure and colloidal properties of four metal oxide nanoparticles were characterized by Fourier transform infrared spectroscopy (FTIR, Bruker Optics GmbH & Co. KG, Ettlingen, Germany), X-ray diffraction (XRD, Bruker AXS GmbH, Karlsruhe, Germany), pH drift method (Mettler-Toledo AG, Greifensee, Switzerland), dynamic light scattering (DLS, Malvern Panalytical Ltd., Malvern, Worcestershire, UK), and electrophoretic light scattering. Among them, hydrodynamic particle size, polydispersity index (PDI), and ζ potential tests are electrokinetic characterization methods commonly used in nanofluids in the study of enhanced oil recovery (EOR) [40]. The pH drift method is used to examine the change in surface charge behavior with pH, and the obtained zero charge condition is regarded as the apparent zero charge point [41].

For DLS and ζ-potential measurements, each sample was measured at least three times independently, with no fewer than 10 sub-cycles per measurement. The results are presented as mean ± standard deviation, and the corresponding error bars represent the standard deviation of the independent measurements. For detailed sample preparation steps, instrument parameters, measurement scope, and data processing methods, please refer to Section S2 of Supplementary Materials.

Scanning electron microscopy (SEM, Carl Zeiss Microscopy GmbH, Jena, Germany) characterization was not performed in the present study. Therefore, the primary morphology, particle shape, surface roughness, and morphology-dependent aggregation structures of the four oxide nanoparticles could not be independently determined. The morphology-related discussion in this work is consequently based on the combined interpretation of XRD, FTIR, hydrodynamic particle size, PDI, and ζ-potential results, rather than on direct microscopic observation.

### 2.3. Rheological, Interfacial, and Adsorption Measurements

The rheological properties of nanofluids were evaluated under different particle concentrations, shear rates, temperatures, and salinities using a stress-controlled rotational rheometer [42,43,44]. The rheological measurement method and concentration-dependent evaluation refer to the methods commonly used in the study of nanoparticle dispersions and nanofluids. Pipe flow experiments were further carried out to characterize the Reynolds number, Darcy friction factor, and pressure gradient response. All viscosity measurements were performed using three independent replicates, and the results are presented as mean ± standard deviation. The error bars in the corresponding figures represent the standard deviation of the replicate measurements. The experimental data were also used to fit conventional and empirical viscosity models to evaluate the concentration-dependent rheological response.

The wettability of the rock was evaluated by measuring the contact angle, and the sandstone sample and the rock core were taken from the same rock material. According to the purpose of the experiment, clean samples and crude-oil-aged samples were used, respectively, and the contact angle was determined at least five positions for each sample. The reported contact angles are presented as mean ± standard deviation, with error bars representing the standard deviation of the measurements at different positions. The interfacial tension of the oil–water system was determined by the pendant-drop method combined with Young–Laplace fitting, and at least three independently prepared droplets were analyzed under each condition. The reported values are presented as mean ± standard deviation, with error bars representing the standard deviation of the independently prepared droplets. The determination of wettability and interfacial tension refers to the methods widely used in evaluating the interaction of nanofluid–rock and oil–water in the EOR system [42,43].

Static and dynamic adsorption experiments were conducted to characterize the interaction between crude oil components and nanoparticles. The adsorption and retention experimental methods are based on mass balance and dynamic transport methods, which have previously been applied to nanoparticle–rock systems in the study of improving the rate of harvest (EOR) [43,44]. At the same time, the adsorption of nanoparticles on sandstone and the dynamic adsorption loss during transport through water-saturated rock cores were determined to evaluate the particle-rock interaction and retention behavior. The oil concentration was determined by n-hexane extraction followed by UV spectrophotometry, and the concentrations of Si, Ti, Al, and Fe were determined by inductively coupled plasma emission spectroscopy when necessary. For detailed experimental schemes, calculation formulas, parameter ranges, and fitting steps, please refer to Sections S3–S5 of Supplementary Materials.

### 2.4. Porous-Media Transport and Core-Flooding Experiments

The transport and retention behavior of nanoparticles in porous media is evaluated using water-saturated sandstone cores. First, the initial permeability is determined using the base fluid, then the nanofluid is injected, and then the base fluid is used for flushing. The migration, retention, and irreversible flow resistance of nanoparticles are characterized by permeability change, pressure response, resistance factor, and residual resistance factor. Static immersion and dynamic injection experiments are carried out separately: the static experiment determines the permeability after 24 h of treatment in the water-saturated rock core with 0.30 wt% nanofluid; the dynamic experiment injects 1.0 PV nanofluid, followed by water flooding.

The core flooding experiment is completed on the high-temperature and high-pressure displacement system. The overall replacement program and the evaluation of the collection rate are consistent with the indoor core replacement method commonly used in the study of nanoparticle-assisted improvement of the collection rate (EOR). The water-saturated rock core is first saturated with crude oil and aged at 60 °C for 7 days. Unless otherwise stated, the reference displacement conditions are: nanoparticle concentration 0.30 wt%, pH 8.0, temperature 70 °C, net confining pressure 1000 psi, back pressure 300 psi, injection rate 1.0 mL min^−1^, nanofluid slug size 1.0 PV, and core permeability of about 500 mD. The initial water flooding continues until the water cut at the outlet end reaches 98%, and then the nanofluid is injected, and the subsequent water flooding is carried out. During the whole displacement process, the oil production, water production, and the pressure difference at both ends of the rock core are recorded.

Further, single-factor experiments are carried out to investigate the influence of hydrodynamic particle size, nanoparticle concentration, pH, nanofluid slug size, temperature, confining pressure, injection rate, soaking time, and core permeability. For detailed parameter ranges, permeability calculation, resistance factor definition, recovery rate calculation, tracer test, and operation steps, please refer to Section S6 of Supplementary Materials.

### 2.5. Oil–Water Separation, Aging, and Recycling Tests

The oil–water separation experiment is carried out by using simulated oily water prepared from crude oil and deionized water. The effects of nanoparticle concentration, initial oil concentration, contact time, treatment volume, temperature, salinity, and ultrasonic treatment on the oil removal effect are investigated. The oil removal efficiency is determined according to the change in the aqueous oil concentration, and the dispersion stability of nanofluids is evaluated by the stability index based on absorbance.

The long-term aging experiment uses 0.30 wt% nanofluid, which is sealed at 25 °C and kept away from light for up to 60 days. At the predetermined aging time, its colloidal, rheological, interfacial, oil removal, and core displacement performance are directly evaluated without another ultrasonic treatment. The recycling performance is evaluated through five consecutive recovery–redispersion cycles. For detailed oily water preparation methods, operating conditions, stability index calculation, nanoparticle recovery steps, and recycling schemes, please refer to Section S7 of Supplementary Materials.

## 3. Results and Discussion

### 3.1. Structural Characteristics and Colloidal Stability of Metal Oxide Nanofluids

**Figure 1:**
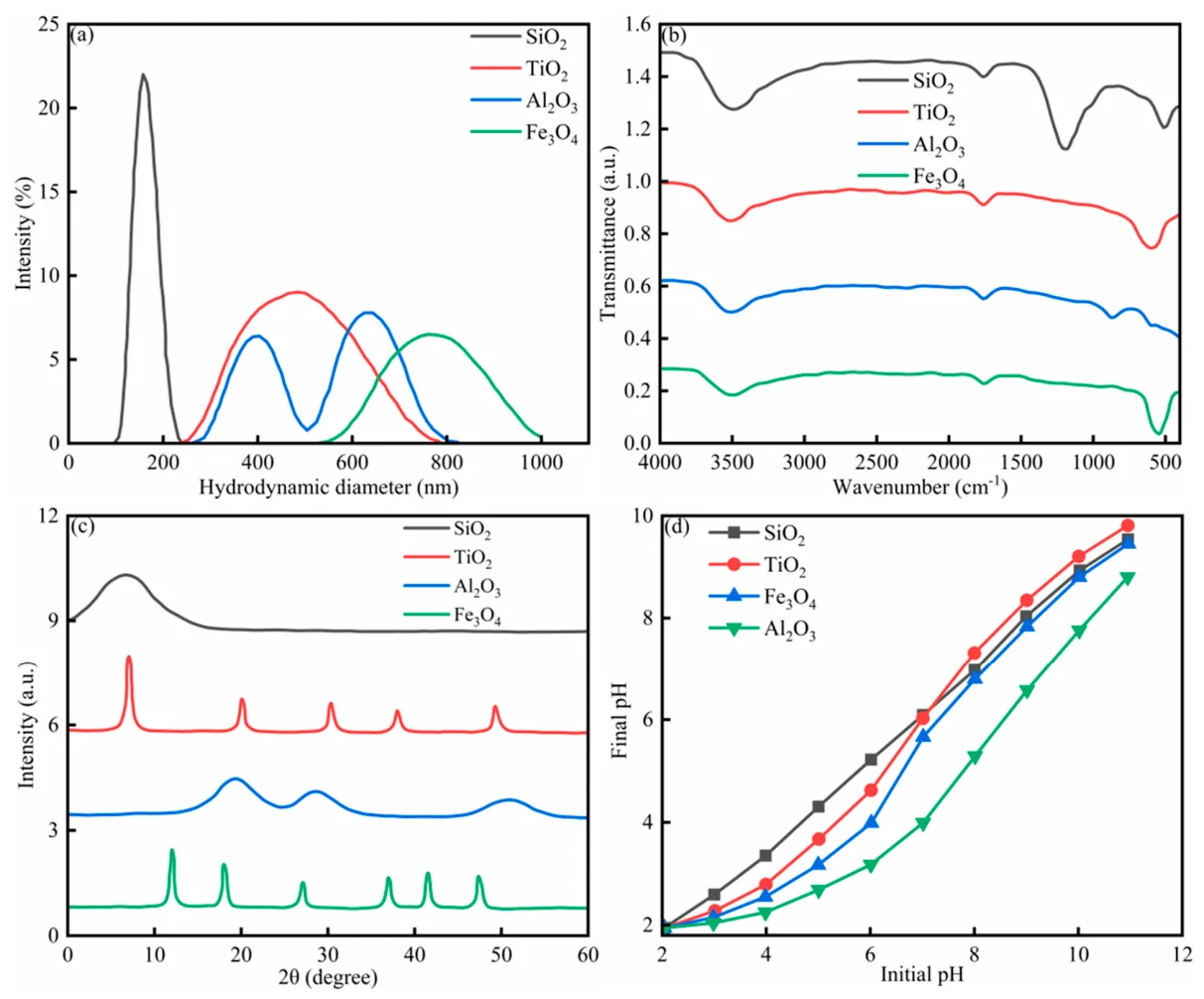
Characterization of the structure and surface properties of different metal oxide nanoparticles. (**a**) Hydrodynamic particle size distribution of SiO_2_, TiO_2_, Al_2_O_3_, and Fe_3_O_4_ nanoparticles in aqueous solution; (**b**) Fourier transform infrared spectra of different nanoparticles; (**c**) X-ray diffraction spectra of different nanoparticles; (**d**) the final pH variation in the nanoparticle suspension under different initial pH conditions.

As shown in Figure 1a, there are significant differences in hydrodynamic particle sizes among different nanoparticles in aqueous solution. Among them, SiO_2_ nanoparticles exhibit a relatively narrow particle size distribution, mainly concentrated around 150 nm, indicating good dispersion ability in the aqueous phase. In addition to particle size and crystal phase, the anisotropy of nanoparticles may also affect interfacial behavior. Morphologically anisotropic particles, such as rod-shaped or flaky particles, may differ from nearly equiaxed particles in terms of exposed surface area, orientation during flow, contact mode with the rock surface, and mechanical retention in the pore throat. Crystallographic anisotropy may also change the density and distribution of hydroxyl groups and active sites on the surface of different exposed crystal surfaces, thus affecting the adsorption and interfacial interaction. Recent studies have shown that the morphology of nanoparticles can significantly affect the zeta potential, wettability changes, interfacial tension, and ultimately affect the crude oil recovery rate. However, this study does not independently regulate the particle shape or crystallographic orientation. Therefore, the differences observed between the four oxide nanofluids should be understood as the results of the joint action of composition, crystal structure, surface chemistry, aggregation state, and effective particle size, and should not be attributed only to the anisotropy of nanoparticles.

The particle size distribution range of TiO_2_ and Al_2_O_3_ nanoparticles gradually expanded, with peaks at about 450 nm and 650 nm, respectively, indicating a certain degree of agglomeration between particles. Fe_3_O_4_ nanoparticles exhibit the largest hydrodynamic particle size, with peak sizes approaching 750 nm, which may be related to strong magnetic interactions between magnetic iron oxide particles. It should be noted that hydrodynamic particle size not only reflects the particle size itself but is also affected by the surface hydration layer and inter-particle attraction. Therefore, larger particle size usually means the particles form a more pronounced aggregated structure under the test environment. Figure 1b further analyzes the surface chemical composition of different nanoparticles. A distinct absorption peak appeared at about 1100 cm^−1^ in SiO_2_ samples, corresponding to the stretching vibration of the Si–O–Si bond, and another near 3400 cm^−1^. The appearance of broadened absorption peaks indicates the presence of a large number of hydroxyl groups on the particle surface. TiO_2_ and Al_2_O_3_ samples mainly exhibit characteristic absorption of metallic oxygen bonds, with the vibration peaks associated with Ti–O and Al–O bonds located in the low wavenumber region, indicating that both oxides maintain intact metal oxide structures. A distinct absorption peak appeared at about 570 cm^−1^ in Fe_3_O_4_ samples, which belongs to Fe–O bond vibrations and is an important structural feature of magnetite. Different oxides have certain hydroxyl groups on their surfaces. These active sites can participate in water molecule adsorption and influence the hydrophilicity and hydrophobic characteristics of nanoparticle surfaces as well as interfacial interactions with crude oil components. The crystal structure of different nanoparticles was further analyzed through XRD (Figure 1c). SiO_2_ shows a wide diffuse diffraction peak rather than an obvious crystal diffraction peak, indicating that it lacks a long-range ordered structure and confirms that it is mainly in an amorphous state. This phenomenon is a typical feature of silicon dioxide with a disordered Si-O-Si network structure. In contrast, TiO_2_ has multiple sharp diffraction peaks, indicating that it has a relatively complete crystal structure. The diffraction characteristics of Al_2_O_3_ are wider and less intense than those of TiO_2_ and Fe_3_O_4_, indicating that it has a lower crystallinity and/or a smaller coherent crystal region, and it cannot be simply considered a completely amorphous structure. This peak width may come from the structural disorder and limited grain growth in transition alumina. Fe_3_O_4_ shows obvious characteristic diffraction peaks at about 30°, 35°, 43°, and 57°, corresponding to the crystalline spinel structure of magnetite. Therefore, the differences between the four oxides in Figure 1c mainly reflect the degree of long-range structural order and the degree of grain development. These structural differences may also further affect the density and distribution of surface hydroxyl groups and active sites, thus affecting their colloidal and interfacial behavior. Differences in crystal structure affect the arrangement of atoms on the surface of nanoparticles and the distribution of active sites, resulting in different interfacial behaviors of different nanomaterials in the aqueous phase. Figure 1d shows the effects of different nanoparticles on the acid-base balance of the solution. As the initial pH increases, the final pH of all nanoparticle suspensions gradually rises, though the magnitude of change varies. Among them, the Al_2_O_3_ system has the lowest final pH under the same initial pH conditions, indicating that its surface hydroxyl groups have strong acid-base responsiveness and can interact with H^+^ or OH^−^ in the solution, causing a more pronounced surface reaction to occur. The trends of SiO_2_, TiO_2_, and Fe_3_O_4_ systems are relatively similar but still exhibit varying degrees of buffering effects. The degree of hydroxylation on the surface of nanoparticles and the electronic properties of the metal centers are important factors affecting their surface acid-base behavior. Different oxides exhibit different protonation and deprotonation capacities due to their different structural compositions.

**Figure 2:**
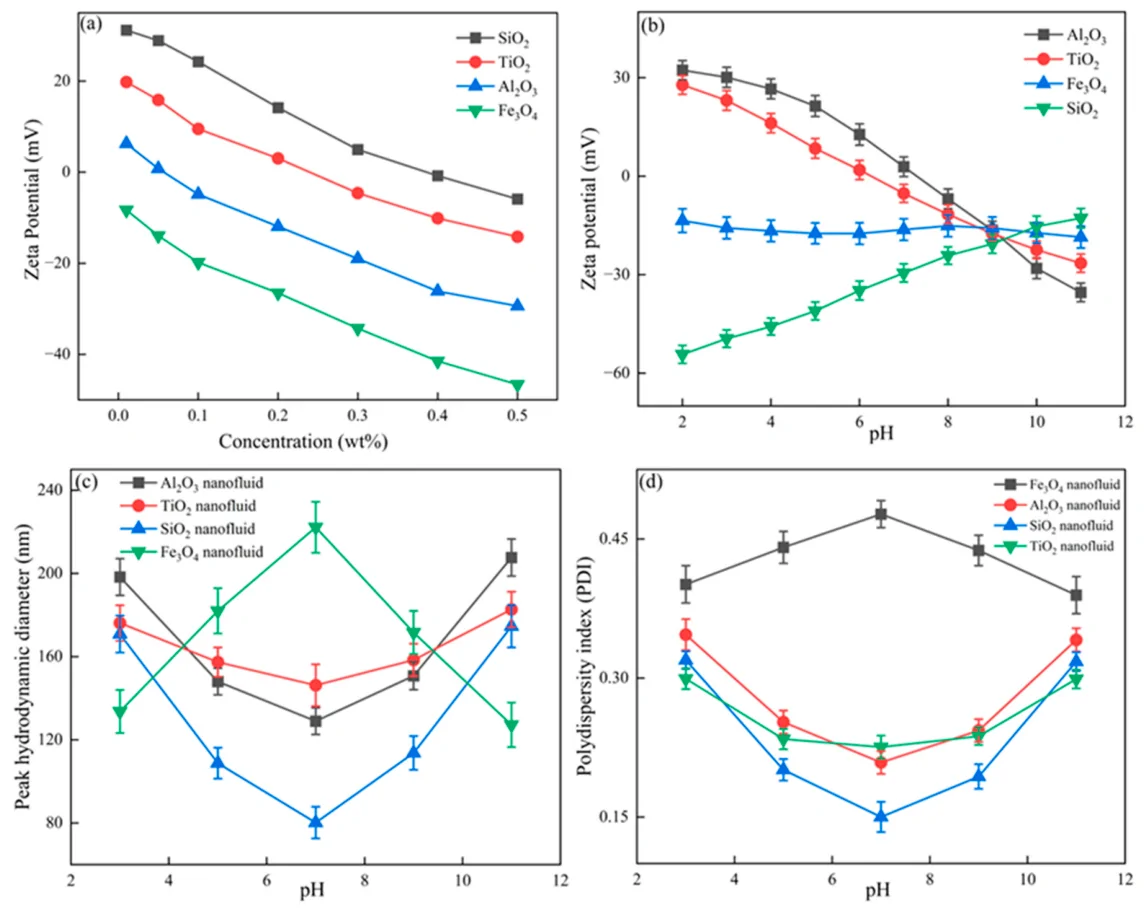
Changes in colloidal stability performance of different metal oxide nanofluids under different concentration and pH conditions. (**a**) The effect of nanoparticle concentration on the potential ζ of SiO_2_, TiO_2_, Al_2_O_3_, and Fe_3_O_4_ nanofluids; (**b**) Al_2_O_3_, TiO_2_, Fe_3_O_4_, and SiO_2_ under different pH conditions and potential changes in ζ nanofluids; (**c**) effect of pH on the hydrodynamic particle size of different nanofluids; (**d**) changes in the polydispersity index of nanofluids under different pH conditions. Error bars represent the standard deviation of the independent replicate measurements.

As shown in Figure 2a, the ζ potential of the four nanofluids changes systematically with the increase in the concentration of nanoparticles, but the magnitude and direction of change vary from material to material. The increase in the concentration of nanoparticles increases the number of oxide surface sites per unit volume, and the average distance between particles decreases. Therefore, the interaction between adjacent electrical double layers becomes more significant, and the protonation/deprotonation of the surface hydroxyl groups and the redistribution of counterions change the electrostatic environment near the slipping plane. These effects may change the measured ζ potential, and in some systems, its absolute value may be reduced through partial compression or shielding of the electrical double layer.

It should be noted that there is no universal linear relationship between the concentration of nanoparticles and the ζ potential. The concentration dependence is determined by the surface chemistry of each oxide, including the acid-base behavior of surface hydroxyl groups, specific ion adsorption, hydration, and interparticle interactions. In this experiment, the ζ potential of SiO_2_ and TiO_2_ showed significant displacement with increasing concentration, while the responses of Al_2_O_3_ and Fe_3_O_4_ were different due to the differences in surface hydroxyl chemistry and interparticle interactions. In particular, the ζ potential observed for Fe_3_O_4_ is increasingly negative, indicating that an increase in the concentration of nanoparticles does not necessarily correspond to a decrease in the absolute value of the ζ potential. Therefore, the results in Figure 2a should be understood as the modification of the electrokinetic environment by concentration, rather than a simple inverse relationship between the mass fraction of nanoparticles and the ζ potential.

Since the ζ potential reflects the contribution of electrostatic interactions to the stability of the colloid, the change in ζ potential caused by the concentration will affect the balance between electrostatic repulsion and attraction between particles. However, the stability of the colloid under high particle concentration cannot be completely predicted by ζ potential alone. At this time, factors such as hydrodynamic particle size, PDI, hydration interactions, and aggregation should also be considered.

Figure 2b further shows that the response of the surface charge of nanoparticles to pH is obviously dependent on the oxide composition. Under acidic conditions, Al_2_O_3_ and TiO_2_ have positive zeta potential, while under the experimental conditions of this study, SiO_2_ and Fe_3_O_4_ are negatively charged. This difference mainly comes from the acid-base behavior of hydroxyl groups on the surface of oxides. The metal-hydroxyl site on the surface can be protonated under low pH conditions, and gradually deprotonated as the pH increases. When the protonated species (≡MOH^2+^) dominates, the particle surface is positively charged; while when the deprotonated species (≡MO^−^) dominates, the particle surface is negatively charged. Therefore, the sign and magnitude of the zeta potential largely depend on the relative relationship between the pH of the solution and the point of zero charge (PZC) or isoelectric point (IEP) of different oxides.

The difference in the surface charge behavior of the four oxides can be further attributed to factors such as metal-oxygen bond properties, surface hydroxyl density and acidity and alkalinity, crystal structure, hydration, and specific ion adsorption, which together affect the protonation/deprotonation equilibrium of the surface site. For Al_2_O_3_ and TiO_2_, the acidic conditions are conducive to the formation of Al–OH^2+^ and Ti–OH^2+^, so it shows a positive ζ potential; as the pH increases, the surface is gradually deprotonated, causing the ζ potential to move in a negative direction. In contrast, the silanol group on the surface of SiO_2_ has a more obvious acidity, so that Si–O^−^ species occupy an important proportion of the wide pH range of this study, thus presenting a negative surface charge. In this study, Fe_3_O_4_ also maintains a negative ζ potential, which may be related to its surface state of hydroxylation/oxidation, ion adsorption, and the hydration structure formed under the current preparation conditions. Therefore, the difference in the ζ potential of the four nanoparticles should be understood as the unique surface acid-base response of different materials, rather than all oxides following the same pH-surface charge change law.

From Figure 2c, it can be seen that the hydrodynamic particle sizes of different nanofluids differ significantly with pH changes. Among them, SiO_2_ nanofluids have the lowest particle size near pH 7, only about 80 nm, but significantly increase under strong acid and alkali conditions, indicating that near-neutral conditions, the electrostatic repulsion between particles is enhanced, facilitating the formation of stable dispersion systems. Al_2_O_3_ nanofluids exhibit large particle size variations across the entire pH range, dropping to about 130 nm near pH 7, but increasing again to above 200 nm at pH 11, indicating that extreme pH conditions may enhance the charge shielding effect on particle surfaces, thereby promoting aggregation. Meanwhile, the variation pattern of the polydispersity index (PDI) was basically consistent with the particle size results (Figure 2d). SiO_2_ nanofluids have the lowest PDI at pH 7, only about 0.15, indicating a relatively uniform particle size distribution, while the Al_2_O_3_ system consistently maintains a high PDI value, indicating more pronounced particle aggregation within it.

### 3.2. Interfacial Regulation and Rheological Behavior of Nanofluids

**Figure 3:**
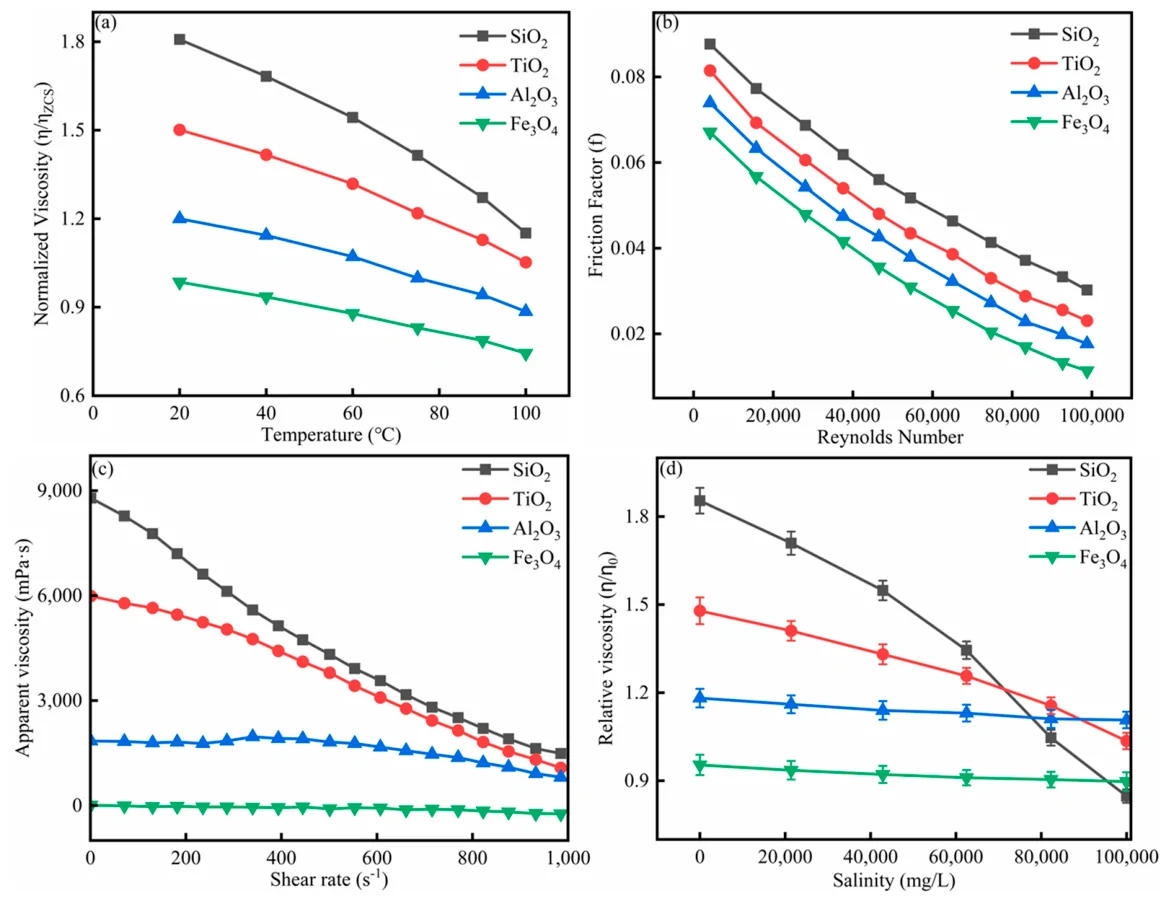
Rheological properties and environmental adaptability analysis of different metal oxide nanofluids. (**a**) The effect of temperature on the normalized viscosity of nanofluids; (**b**) the effect of Reynolds number on the friction factor of nanofluids; (**c**) the effect of shear rate on the apparent viscosity of nanofluids; (**d**) the effect of salinity on the relative viscosity of nanofluids. Error bars represent the standard deviation of the independent replicate measurements.

As shown in Figure 3a, as the temperature increases from 20 °C to 100 °C, the normalized viscosity of all four nanofluids gradually decreases, but the degree of decrease varies significantly among different systems. Among them, SiO_2_ nanofluids maintained the highest viscosity level across the entire temperature range, dropping from about 1.8 to 1.15; TiO_2_ systems decreased from about 1.5 to 1.05; Al_2_O_3_ systems decreased from about 1.2 to 0.9; the Fe_3_O_4_ system showed relatively small variations, dropping from about 0.98 to 0.75. As temperature increases, molecular movement in the continuous phase increases, and internal friction in the liquid phase weakens, so all systems show a declining viscosity trend. The SiO_2_ system still maintains high viscosity, indicating that its hydroxyl structure on the particle surface can form strong hydration reactions with water molecules, and a certain degree of particle–liquid interaction can be maintained under heating conditions. In contrast, the Fe_3_O_4_ system has a lower viscosity, indicating that its particle structure contributes little to fluid viscosity. The effect of changes in Reynolds number on the friction resistance factor of nanofluids is shown in Figure 3b. As the Reynolds number increases from a low value to 100,000, the friction factor of each nanofluid gradually decreases. SiO_2_ nanofluids maintained the highest friction factor throughout the test range, dropping from about 0.087 to 0.030; the TiO_2_, Al_2_O_3_, and Fe_3_O_4_ systems decreased to approximately 0.023, 0.018, and 0.011, respectively. The reduction in friction factor mainly comes from the enhanced inertial effect after increasing flow velocity, which lowers the contribution ratio of viscous resistance to total flow resistance. Differences among different nanofluids are related to system viscosity and particle dispersion state. SiO_2_ nanofluids with higher viscosity exhibit greater flow resistance, while Fe_3_O_4_ systems, due to lower viscosity and different particle interaction modes, have relatively weaker friction response. Figure 3c further investigates the effect of shear rate on apparent viscosity. As the shear rate increases from 0 to 1000 s^−1^, SiO_2_ and TiO_2_ nanofluids exhibit obvious shear-thinning behavior. Among them, the apparent viscosity of the SiO_2_ system decreased from about 8800 mPa·s to about 1500 mPa·s, and the TiO_2_ system decreased from about 6000 mPa·s to about 1200 mPa·s. The Al_2_O_3_ system maintains about 2000 mPa·s during the low-shear phase, then slowly decreases; the Fe_3_O_4_ system has the lowest overall viscosity and does not vary significantly with shear rate. Shear thinning indicates that nanoparticles may form certain spatial structures under static or low shear conditions, but as shear increases, these structures gradually break down, and the particle arrangement tends toward the flow direction, thereby reducing flow resistance. The significant viscosity reduction in SiO_2_ and TiO_2_ systems indicates strong internal particle interactions and greater sensitivity to external shear effects. The effect of salinity changes on the relative viscosity of nanofluids is shown in Figure 3d. As salinity increased from 0 to 100,000 mg/L, viscosity decreased to varying degrees across different systems. Among them, the SiO_2_ nanofluids showed the most significant changes, dropping from about 1.85 to 0.85; the TiO_2_ system decreased from about 1.48 to 1.05; and the Al_2_O_3_ system basically stayed around 1.1; the Fe_3_O_4_ system showed minimal change. In high-salinity environments, a large amount of electrolyte ions entering the system compresses the double-layer structure on the nanoparticle surface, reducing electrostatic repulsion between particles and making them more prone to aggregation, thereby reducing system viscosity. SiO_2_ nanofluids are sensitive to salinity changes, indicating that their stable dispersion mainly depends on surface charges. In contrast, the Al_2_O_3_ and Fe_3_O_4_ systems show less variation, possibly related to their surface charge characteristics and inter-particle interaction patterns. Comprehensive rheological test results reveal that different nanoparticles have significant differences in their ability to regulate fluid structure. SiO_2_ nanofluids have high viscosity and strong shear response, effectively increasing the flow resistance of replacement fluids and improving the flow ratio; The TiO_2_ system maintains relatively stable rheological performance under different environmental conditions; The Al_2_O_3_ and Fe_3_O_4_ systems have weak responses to changes in temperature, shear, and salinity. These results indicate that the surface structure of nanoparticles and interactions between particles are important factors determining the rheological behavior of nanofluids, and changes in rheological performance further affect their migration capacity and oil displacement in reservoirs.

As shown in Figure S2, model results indicate that the viscosity of nanofluids is significantly concentration-dependent, but different particles have varying viscosity efficiency due to differences in surface properties and dispersion states. The similarity trend between experimental data and model predictions indicates that the model can effectively describe the impact of nanoparticle concentration on system viscosity changes. From the viscosity control results, it can be seen that different nanoparticles have varying degrees of influence on fluid rheological properties. SiO_2_ nanofluids have the most pronounced viscosity enhancement effect, effectively increasing the viscosity of replacement fluids and helping to improve oil–water flow matching; the TiO_2_ system has good viscosity enhancement while maintaining certain flowability; the Al_2_O_3_ and Fe_3_O_4_ systems have relatively weak viscosity enhancement effects. Therefore, increasing the concentration of nanoparticles can enhance the effect of rheological regulation, but this benefit must be weighed against the increasing tendency of aggregation and injection resistance. Therefore, in enhanced oil extraction applications, it is necessary to reasonably select nanoparticle types and addition concentrations based on reservoir seepage conditions to achieve a balance between viscosity regulation capability and injection performance.

**Figure 4:**
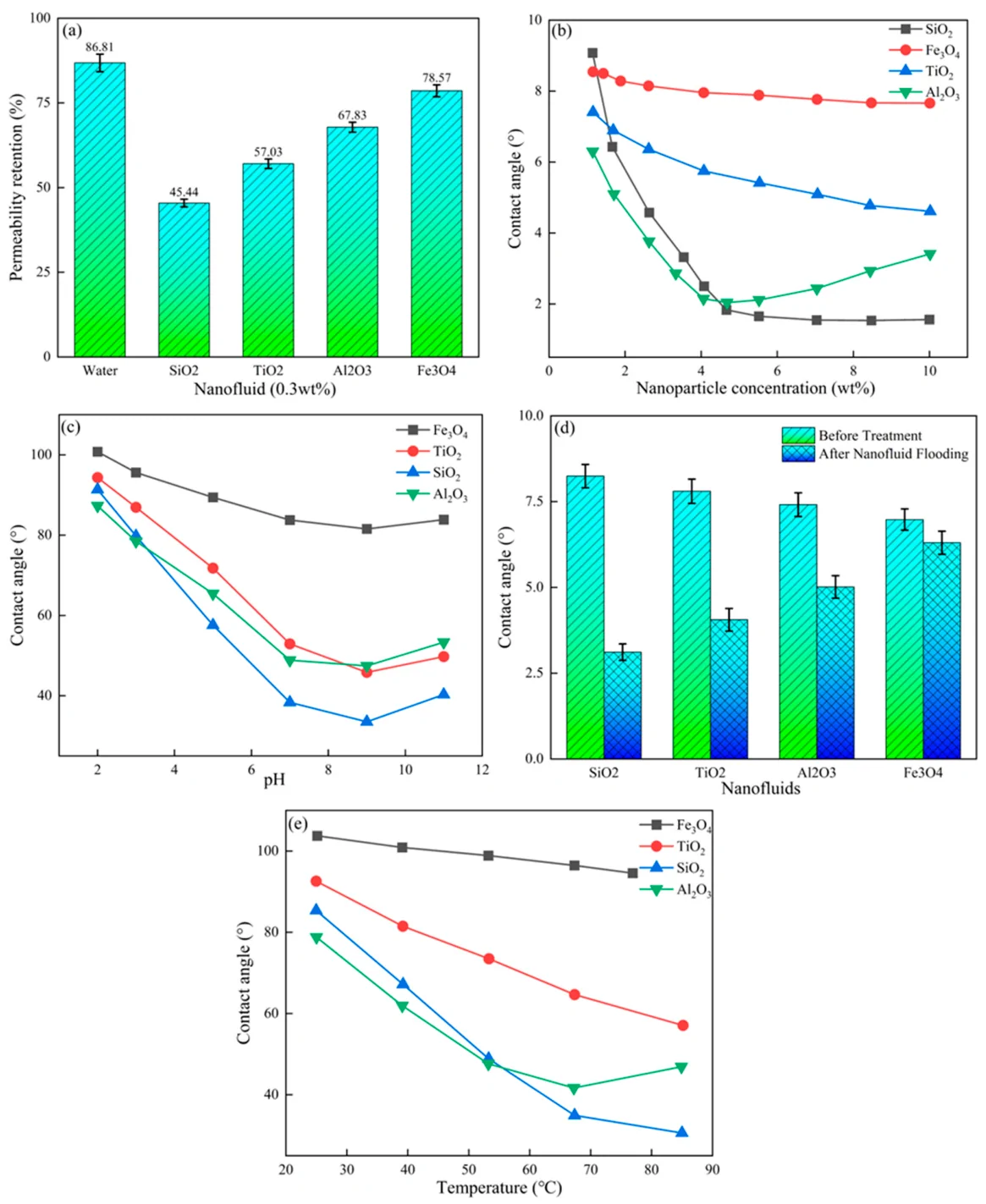
Changes in rock permeability and wettability characteristics after the action of different metal oxide nanofluids. (**a**) Core permeability retention rate after different nanofluid treatments; (**b**) the effect of nanoparticle concentration on the angle of contact with the rock; (**c**) the effect of solution pH on the angle of contact with the rock; (**d**) changes in rock contact angle before and after nanofluid displacement; (**e**) the effect of temperature on the angle of contact with rocks. Error bars represent the standard deviation of the independent replicate measurements.

As shown in Figure 4a, under the same concentration (0.30 wt%) conditions, there are significant differences in core permeability retention after different nanofluid treatments. Under water flooding, the permeability retention rate was 86.81%, while all nanofluids decreased after treatment, with SiO_2_ systems having the lowest at 45.44%; the TiO_2_, Al_2_O_3_, and Fe_3_O_4_ systems were 57.03%, 67.83%, and 78.57%, respectively. The decrease in permeability is mainly related to the adsorption and local retention of nanoparticles on the pore surface. SiO_2_ nanoparticles, due to their high surface hydroxyl content, have a strong interaction with the mineral surface, making them easier to adhere to pore walls and thereby increasing flow resistance. In contrast, the Fe_3_O_4_ system maintains a higher permeability, indicating that its particles have a lower degree of adsorption in the pores and less impact on the flow channel. These results indicate that the effect of nanoparticles on pore structure is related to surface activity and migration ability; strong adsorption helps alter interface properties but may also reduce seepage capacity.

Figure 4b shows that the effect of nanoparticle concentration on the surface wettability of sandstone is obviously material-dependent. For SiO_2_, TiO_2_, and Fe_3_O_4_, as the concentration of nanoparticles increases, the contact angle generally declines or gradually stabilizes. In contrast, Al_2_O_3_ showed obvious non-monotonic changes: the contact angle first decreased, reached the lowest value near the medium concentration, and then rebounded slightly at higher concentrations.

At low to medium Al_2_O_3_ concentrations, increasing the concentration of nanoparticles can improve the adsorption probability of particles on the surface of sandstone and promote the formation of a particle-covering layer rich in hydroxyl groups and hydration structures, which is conducive to the spread of the aqueous phase on the surface of the rock, thus reducing the contact angle. However, when the concentration of Al_2_O_3_ increases further, enhanced particle–particle interactions and aggregation may lead to uneven deposition or multilayer particle deposition on the rock surface. This kind of aggregate deposition may partially obscure the hydrophilic active sites and, at the same time, increase the surface non-uniformity and three-phase contact line pinning effect, thus weakening the further wetting modification effect. Therefore, if the concentration of Al_2_O_3_ continues to increase after the contact angle reaches the lowest value, the contact angle may increase again.

This explanation is consistent with the wider particle size distribution and higher PDI of Al_2_O_3_ nanofluids, indicating that it has a more obvious tendency toward agglomeration than SiO_2_. Therefore, the results for Al_2_O_3_ indicate that there may be a suitable concentration range: within this range, the degree of particle coverage is sufficient to enhance the moisture on the surface of the rock, and at the same time, it will not weaken the wetting modification effect due to excessive aggregation and non-uniform deposition. Since this study does not directly characterize the surface morphology of the treated rock, the multilayer sedimentation and surface non-uniformity proposed here should be used as a mechanism explanation, not direct experimental evidence.

The effect of solution pH on the contact angle is shown in Figure 4c. As pH increases from 2 to 11, the contact angles of SiO_2_, TiO_2_, and Al_2_O_3_ systems all decrease significantly, while the changes in Fe_3_O_4_ systems are relatively limited. Among them, the SiO_2_ system reaches a minimum contact angle near pH 9, about 34°; the TiO_2_ and Al_2_O_3_ systems are reduced to about 45° and 47°, respectively. Changes in pH regulate the form of hydroxyl groups present on the surface of nanoparticles. In acidic environments, the hydroxyl group on the surface undergoes protonation, reducing the surface charge of particles and weakening the interaction between particles and rocks; as pH increases, hydroxyl deprotonation increases, increasing the negative charge on the particle surface, which facilitates stable dispersion and adsorption on the rock surface. Therefore, a suitable alkaline environment can enhance the nanofluid’s ability to regulate wettability. Figure 4d compares the changes in rock contact angles before and after nanofluidic treatment. After nanofluid displacement, the contact angles of all systems significantly decreased, with the largest variation after SiO_2_ treatment, dropping from about 8.5° to about 3°; the TiO_2_, Al_2_O_3_, and Fe_3_O_4_ systems also showed varying degrees of decline. These results indicate that during the replacement process, nanoparticles can enter areas near the rock surface and alter the original wetting state through adsorption. After wettability shifts to hydrophilicity, the aqueous phase more easily enters the crude oil coverage area, reducing the stability of crude oil attachment on the rock surface, which is beneficial for residual oil release. As the temperature rises from 25 °C to 85 °C, the contact angles after treatment with different nanofluids tend to decrease. Among them, the SiO_2_ system was reduced from about 85° to 31°, the TiO_2_ system from about 92° to 57°, the Al_2_O_3_ system from about 78° to 47°, and the Fe_3_O_4_ system showed relatively minor changes. As the temperature rises, molecular motion intensifies, and the diffusion rate of nanoparticles at the oil–water interface and the rock surface increases, resulting in more complete contact between the particles and the mineral surface. Therefore, under high-temperature conditions, the effect of nanofluids on rock wettability is enhanced.

### 3.3. Transport Behavior and Adsorption Mechanism in Porous Media

**Figure 5:**
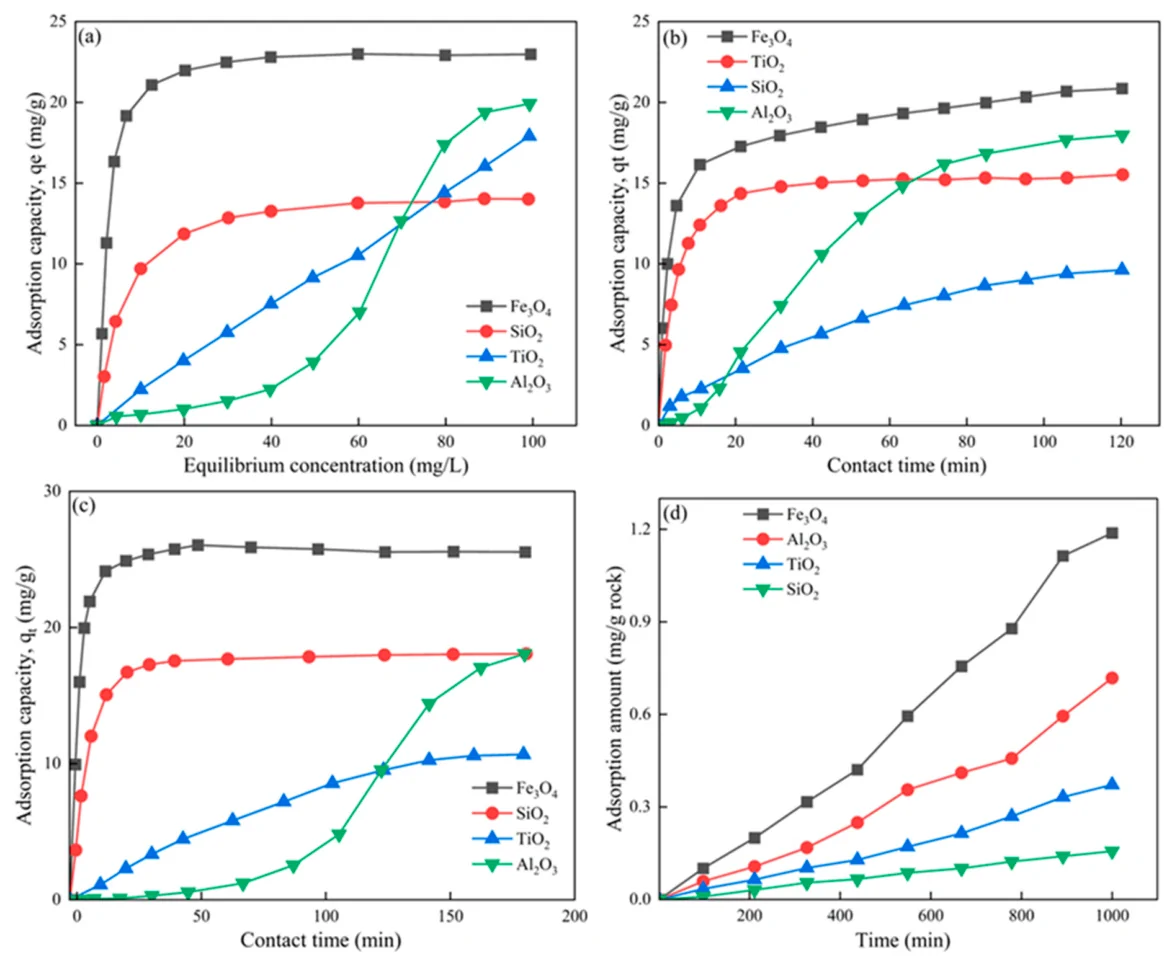
The kinetic and equilibrium behavior of different metal oxide nanoparticles adsorbing crude oil components. (**a**) The effect of equilibrium concentration on the adsorption capacity of nanoparticles; (**b**) the effect of adsorption time on dynamic adsorption capacity; (**c**) equilibrium adsorption capacity changes under different contact times; (**d**) changes in adsorption amount per unit rock mass at different adsorption times.

As shown in Figure 5a, as the equilibrium concentration increases, the adsorption capacities of all four nanoparticles gradually increase and tend to stabilize under high concentration conditions. Fe_3_O_4_ nanoparticles exhibit the highest adsorption capacity, reaching about 23 mg/g near equilibrium concentrations of 100 mg/L; Al_2_O_3_ and TiO_2_ systems reach approximately 20 mg/g and 18 mg/g, respectively; the SiO_2_ system has the lowest adsorption capacity, with a stable value of about 14 mg/g. The adsorption capacity increases with concentration, mainly because at low concentrations, many unoccupied adsorption sites exist on the particle surface, allowing crude oil components to quickly bind to the particle surface. As the concentration continues to increase, the active sites on the surface of particles are gradually occupied, and the adsorption process is limited by surface capacity, so the growth of adsorption volume tends to slow down. Differences among different nanomaterials indicate that the particle surface structure and number of active sites directly affect the adsorption capacity of crude oil components. Figure 5b further analyzes the effect of adsorption time on dynamic adsorption capacity. The results showed that each nanoparticle exhibited two processes: a rapid adsorption phase and a slow equilibrium stage. During the initial adsorption phase, the adsorption capacity of Fe_3_O_4_ nanoparticles rapidly increases, reaching a high level in a short time, then slowly increases and stabilizes, eventually exceeding 20 mg/g. The TiO_2_ system also exhibited rapid adsorption, ultimately stabilizing at about 15 mg/g; the adsorption capacity of the Al_2_O_3_ system increases over time, reaching about 18 mg/g at 120 min; the SiO_2_ system grows relatively slowly, eventually reaching about 10 mg/g. The rapid adsorption stage is mainly related to the external mass transfer process of crude oil components to the particle surface, while the slowdown in later growth is due to gradual reduction in adsorption sites and increased internal diffusion resistance. The relatively high initial adsorption rate of Fe_3_O_4_ indicates strong adsorption activity on its surface. The changes in adsorption capacity under different contact times are shown in Figure 5c. As the contact time increases, the adsorption capacity of all systems gradually increases, but the time required to reach a stable state varies. After about 40 min, Fe_3_O_4_ nanoparticles basically reached adsorption equilibrium, with a maximum adsorption capacity of about 25 mg/g; the SiO_2_ system reached about 18 mg/g in a relatively short time and remained stable; the adsorption capacity of the TiO_2_ system continues to increase, reaching equilibrium after 150 min; the Al_2_O_3_ system shows a clear increasing trend under prolonged exposure, eventually reaching about 18 mg/g. These results indicate that different nanoparticles interact with crude oil components to varying degrees, and the chemical properties of the particle surface determine the adsorption rate and final capacity. Figure 5d further compares the changes in adsorption capacity of nanoparticles acting on the rock surface. As the adsorption time increases, the adsorption capacity per unit rock mass gradually increases, with the Fe_3_O_4_ system showing the most significant increase, reaching about 1.2 mg/g at 1200 min; the Al_2_O_3_ system is about 0.7 mg/g; the TiO_2_ system is about 0.4 mg/g; the SiO_2_ system has the lowest concentration, about 0.15 mg/g. A higher amount of rock surface adsorption indicates a strong interaction between Fe_3_O_4_ particles and mineral surfaces, which can form noticeable surface retention. However, stronger adsorption may also reduce the movement ability of particles within the pores, limiting their range of action. In contrast, although SiO_2_ has a lower adsorption capacity, combined with the aforementioned flooding oil experiments, its good migration ability and interfacial regulation enable it to operate over a larger range.

The adsorption behavior of nanoparticles in reservoir pores determines their migration distance and effective range of action, and also affects the ability to regulate flow resistance during subsequent displacement processes. As shown in Figure S3, the retention characteristics of different nanomaterials in porous media, the effects of temperature, flow rate, particle concentration, and particle size on dynamic adsorption loss and residual resistance were examined. From the adsorption and resistance regulation results, it can be seen that different nanoparticles exhibit significant differences in behavior in porous media. Fe_3_O_4_ nanofluids have strong adsorption and retention capacity, can create high residual resistance, which helps regulate the flow ratio, but excessive retention may restrict particle movement to deeper areas. SiO_2_ nanofluids exhibit lower adsorption loss and better mobility, which is more conducive to expanding their range of action. The Al_2_O_3_ and TiO_2_ systems lie between the two, and their adsorption behavior is influenced by both particle size and surface properties. Therefore, in the process of nanofluid-intensified oil extraction, it is necessary to coordinate particle adsorption capacity and migration performance so that nanoparticles can remain effectively in pores while achieving broad interfacial regulation.

**Figure 6:**
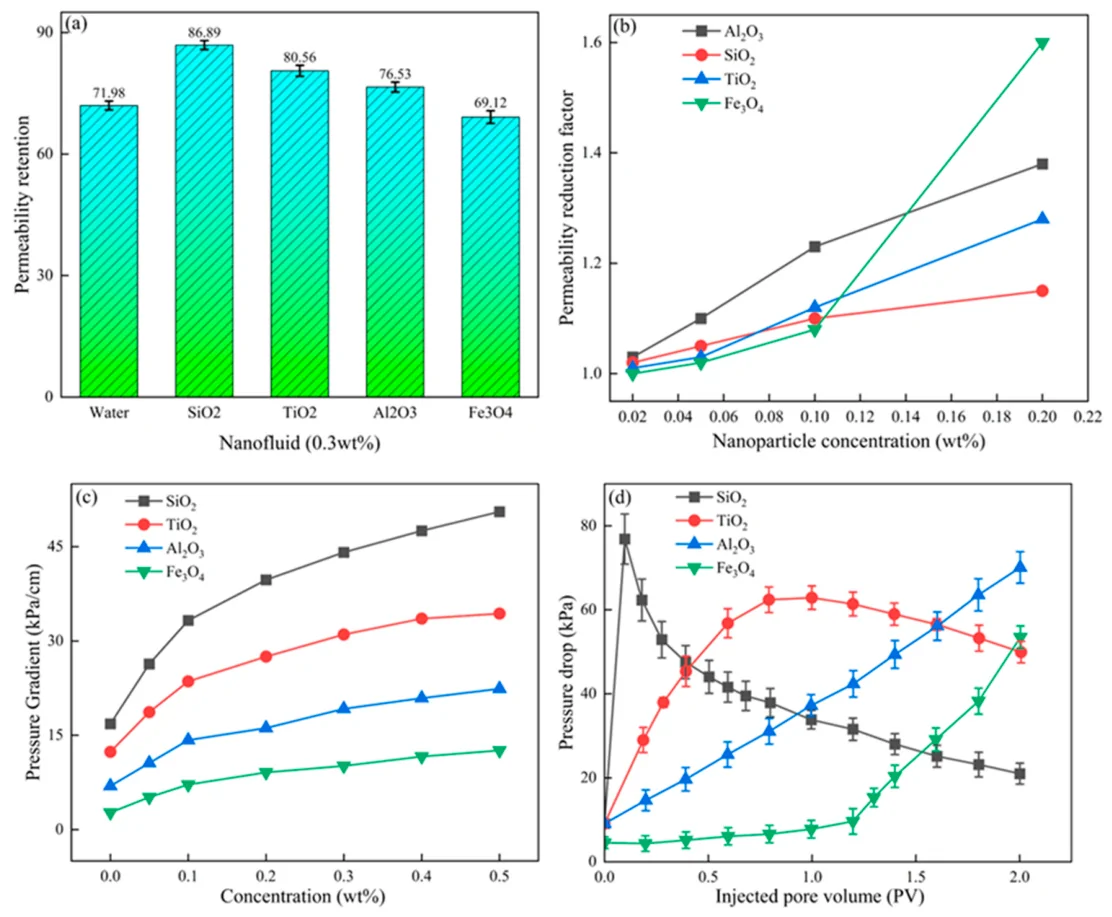
Effects of different metal oxide nanofluids on the permeability of porous media. (**a**) Core permeability retention rate after different nanofluid treatments; (**b**) the effect of nanoparticle concentration on the permeability reduction coefficient; (**c**) the effect of nanoparticle concentration on displacement pressure gradient; (**d**) the effect of injection pore volume on changes in displacement pressure drop. Error bars represent the standard deviation of the independent replicate measurements.

As shown in Figure 6a, under the same concentration (0.30 wt%) conditions, different nanofluids exhibited significant differences in their ability to maintain core permeability. Under water flooding, the permeability retention rate was 71.98%, while after adding nanoparticles, all showed varying degrees of change, with the SiO_2_ system being the highest, reaching 86.89%; the TiO_2_, Al_2_O_3_, and Fe_3_O_4_ systems were 80.56%, 76.53%, and 69.12%, respectively. These results indicate that after nanoparticles enter the pores, they do not simply cause pore blockage, but are instead affected by particle size, dispersion state, and surface acting capacity. SiO_2_ nanoparticles have good dispersion stability and are not prone to large-scale aggregation in pores, thus maintaining high seepage capacity. Fe_3_O_4_ systems have the lowest permeability retention, possibly due to strong interactions between magnetic particles. After particles aggregate, they tend to remain at the pore throat position, increasing flow resistance. Figure 6b further analyzes the effect of particle concentration on the permeability reduction coefficient. As the nanoparticle concentration increased from 0.02 wt% to 0.20 wt%, the permeability reduction coefficient for all systems gradually increased, with the Fe_3_O_4_ system showing the most significant increase, from about 1.0 to 1.60; the Al_2_O_3_ system reaching about 1.38; the TiO_2_ system increasing to about 1.28; the SiO_2_ system exhibiting the least significant change, increasing only to about 1.15. As particle concentration increases, the number of nanoparticles entering the pore space rises, and the contact frequency between particles and the rock surface rises, enhancing adsorption and retention effects. Therefore, the permeability reduction coefficient increases with concentration. Differences among different materials indicate that particle concentration is not the sole determining factor; particle surface properties and aggregation tendencies also control their impact on pore structure. The high reduction coefficient of Fe_3_O_4_ indicates its strong pore regulation ability, but excessive retention may reduce particles’ ability to migrate into deeper pores. The effect of nanoparticle concentration on pressure gradient changes is shown in Figure 6c. As the concentration increases from 0 to 0.5 wt%, the pressure gradients for different nanofluid displacements show an increasing trend. Among them, the SiO_2_ system showed the most significant change, increasing from about 17 kPa/cm to 50 kPa/cm; the TiO_2_ system increased from about 12 kPa/cm to 34 kPa/cm; the Al_2_O_3_ system reached about 23 kPa/cm; the Fe_3_O_4_ system only increased to about 13 kPa/cm. An increase in pressure gradient indicates that the addition of nanoparticles enhances the resistance the fluid experiences when passing through the pore medium. On the one hand, particles adsorbed onto the hole wall change the local flow cross-section. On the other hand, the interaction between particles and fluids increases energy loss during the flow process. The SiO_2_ system exhibits a high pressure gradient, indicating that it can effectively alter the fluid flow state within the pores, which helps improve flow matching during displacement. Figure 6d shows the variation patterns of pressure drop of different nanofluids during the increase in PV injection. Different systems exhibit distinctly different stress response characteristics. SiO_2_ nanofluids exhibit a high pressure response during the initial injection stage, which gradually decreases as the injection process progresses; the pressure drop in the TiO_2_ system continues to rise with PV and has reached a high level near 1.0 PV; the trend of changes in the Al_2_O_3_ system is similar to that of TiO_2_, but overall stress levels are relatively low; the pressure variation in the Fe_3_O_4_ system is minimal in the early stages, but gradually increases in the later stages as the injection volume increases. This difference reflects the different migration processes of nanoparticles in porous media. SiO_2_, due to its strong dispersion ability, can quickly enter pores and interact with pore walls; Fe_3_O_4_, due to stronger interparticle interaction, gradually becomes retained as the injection time increases, leading to increased pressure later on.

### 3.4. Enhanced Oil Recovery Performance Under Reservoir Conditions

In the process of nanofluid oil displacement, the particle size also affects the surface activity, adsorption/retention behavior, and accessibility of particles to pore throats, so it is an important parameter. It can be seen from Figure S4a that the recovery rate of the four nanofluids changes non-monotonically with the hydrodynamic particle size: when the particle size is small, the recovery rate increases with the increase in the particle size; it reaches the highest value at the medium particle size; and it begins to decline after continuing to increase. This shows that the optimal particle size cannot be determined simply by pursuing the maximum specific surface area or the minimum particle size.

The adsorption results in Figure S2d further explain the above phenomenon with the change in particle size. Particles with smaller particle sizes show greater dynamic adsorption losses, which are related to their larger accessible surface area and more frequent contact with the rock surface. Although this kind of contact may be conducive to interface modification, excessive near-surface adsorption will reduce the number of particles that can be transported deep. On the contrary, it is difficult for too large hydrodynamic aggregates to enter the narrow pore throat, and they are more prone to mechanical retention, which will also limit their effective transport distance. Therefore, under the sandstone conditions covered in this research, medium hydrodynamic particle size is more conducive to achieving a balance between interfacial activity, retention, and deep transport.

The importance of nanoparticle size in EOR has been confirmed in previous studies. Abe et al. examined SiO_2_ nanofluids of different particle sizes through wettability experiments and core flooding experiments, and found that nanoparticles with smaller particle sizes can cause stronger wettability changes and obtain higher additional oil recovery. In contrast, this study observes that the crude oil recovery rate changes non-monotonically with hydrodynamic particle size. This difference may be due to the fact that this study considers the effective hydrodynamic size of dispersed particles and aggregates in a wider particle size range, and the influence of particle retention on the rock surface and pore throat accessibility becomes more prominent in this range. Therefore, the impact of particle size on EOR should be jointly evaluated in combination with the aggregation state and the transport in the porous medium, and cannot be judged based on the particle size alone.

**Figure 7:**
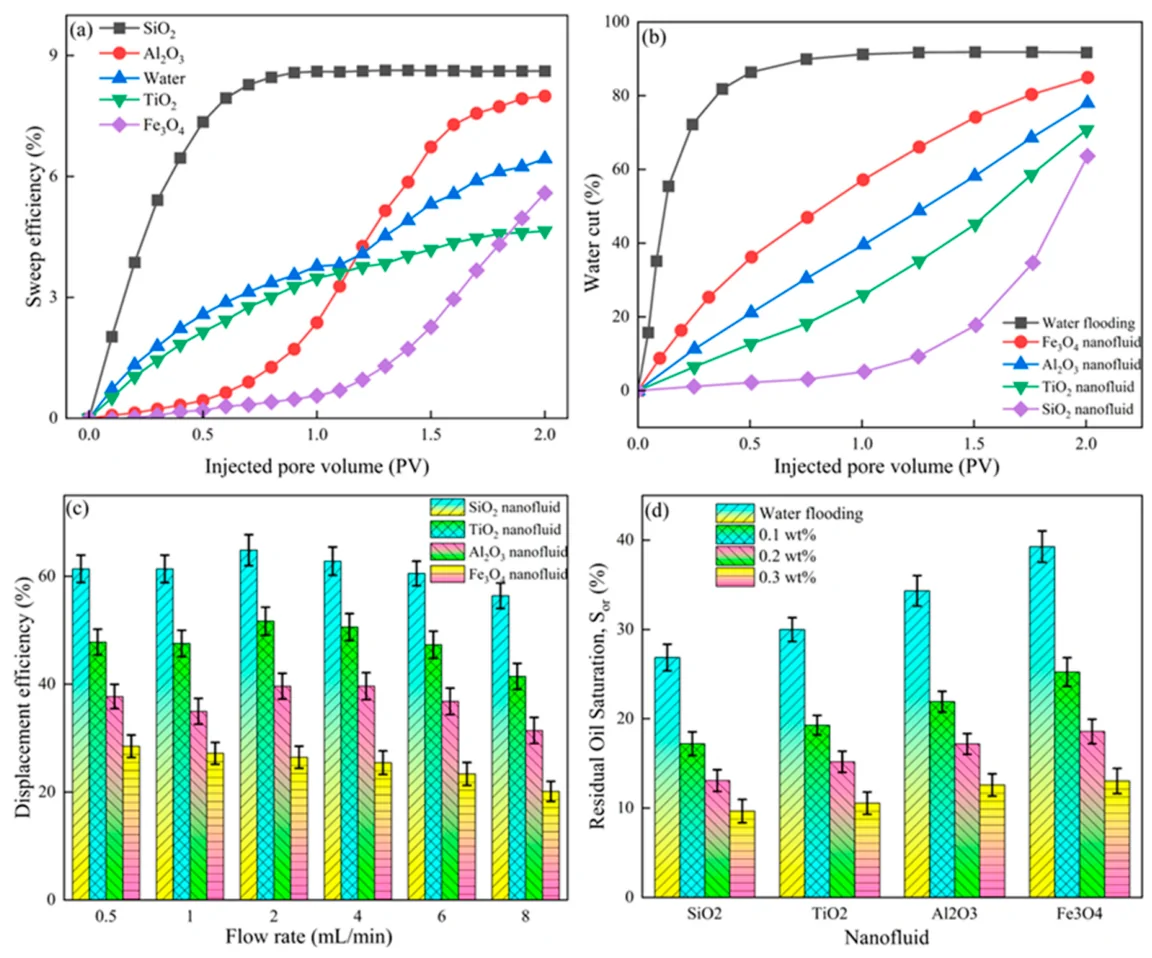
Dynamic responses and oil displacement effects of different metal oxide nanofluids during the process of displacement of porous media. (**a**) The effect of injected pore volume on the wave-reach efficiency of nanofluids; (**b**) changes in moisture content during the injection process of different replacement systems; (**c**) changes in the efficiency of nanofluid displacement under different flow rate conditions; (**d**) changes in residual oil saturation after different nanofluids. Error bars represent the standard deviation of the independent replicate measurements.

As shown in Figure 7a, as the injected pore volume (PV) increases, the wave efficiency of different nanofluidic systems gradually improves, but there are significant differences in growth rate and final stabilization value. During the water flooding phase, the ripple efficiency improves rapidly, reaching a high level under low injection rate conditions, followed by stabilization. In contrast, nanofluidic systems grow more slowly in the initial stage, but their ripple capacity gradually increases as the injection process continues. Among them, SiO_2_ nanofluids showed a rapid growth trend, basically stabilizing after about 0.8 PV, with a final spillover efficiency of about 8.5%; TiO_2_ and Al_2_O_3_ nanofluids reached approximately 5.0% and 8.0%, respectively; the initial increase in Fe_3_O_4_ nanofluids was relatively slow, gradually increasing under high PV injection conditions, eventually reaching about 5.5%. This change indicates that after nanoparticles enter the pore medium, they undergo certain migration processes, and as particles redistribute within the pore space, their regulatory effect on the flow path gradually becomes apparent. The higher wave efficiency of SiO_2_ systems may be related to their smaller particle size distribution and better dispersion stability, allowing them to enter deeper pore regions and improve the displacement solution’s spread range. Changes in moisture content during injection of different replacement systems are shown in Figure 7b. During water flooding, water content rises rapidly, reaching a high level under low PV injection conditions, indicating that the water phase easily breaks through the dominant channel quickly, leading to decreased replacement efficiency. In contrast, nanofluids can significantly reduce the rate of water content rise, with Fe_3_O_4_ maintaining the lowest water content throughout the injection process, about 64% at 2.0 PV; the TiO_2_ and Al_2_O_3_ systems reach approximately 70% and 78%, respectively. Although the water content of SiO_2_ nanofluids increases later, it remains lower than that of the hydroxyl process. The law of water content variation shows that after nanoparticles enter the pores, local flow resistance is altered, limiting the dominant flow of replacement fluids in the high-osmotic channel and thus delaying water transmigration. The differences among various nanofluids mainly stem from differences in adsorption between particles and pore walls, particle migration capacity, and ability to generate flow resistance. Figure 7c further investigates the effect of flow velocity on replacement efficiency. The results show that within the range of 0.5~8 mL/min, the displacement efficiency of each nanofluid system changes little, indicating that nanoparticles can maintain stable action within a certain flow rate range. Among them, SiO_2_ nanofluids maintained the highest displacement efficiency under different flow rate conditions, approximately 60~65%; TiO_2_ systems should be maintained at around 45~50%; Al_2_O_3_ and the Fe_3_O_4_ systems are lower than the two systems mentioned above. As the flow rate increases, displacement efficiency does not significantly improve but instead fluctuates, indicating that excessively high flow rates may shorten the interaction time between nanoparticles and residual oil, limiting interface regulation. Therefore, the nanofluidic oil displacement process is affected not only by injection speed but also by the retention time of particles in pores and the strength of interfacial interactions. The changes in residual oil saturation after different nanofluids are shown in Figure 7d. Compared to water flooding, nanofluids can reduce residual oil content in the core, with the SiO_2_ system showing the most significant reduction effect. As SiO_2_ concentration increases, residual oil saturation further decreases from about 17% to about 13%; TiO_2_ and Al_2_O_3_ systems were reduced to about 18% and 17%, respectively; the Fe_3_O_4_ system has the highest residual oil saturation, about 25%. The reduction in residual oil is mainly related to the regulation of the oil–water interface by nanoparticles. After adsorbing onto the rock surface, nanoparticles alter the surface wetting state and weaken the adhesion between crude oil and mineral surfaces, allowing some difficult-to-flow residual oil to re-enter the replacement process.

As shown in Figure S5, the oil displacement effect of nanofluids is jointly influenced by material properties and reservoir environment. SiO_2_ nanofluids exhibit high oil displacement efficiency under different temperature, pressure, flow rate, and permeability conditions, which is related to their good dispersion stability and interfacial regulation capabilities; TiO_2_ and Al_2_O_3_ systems have some strengthening effects, but their improvement is limited by particle migration and interfacial interaction capabilities. Although Fe_3_O_4_ systems have strong adsorption capacity, their particle aggregation tendency may limit their effective propagation in pores. Therefore, in practical applications, it is necessary to select appropriate nanomaterials based on reservoir temperature, pressure, and pore structure characteristics to achieve a balance between particle migration capacity and interface regulation.

**Figure 8:**
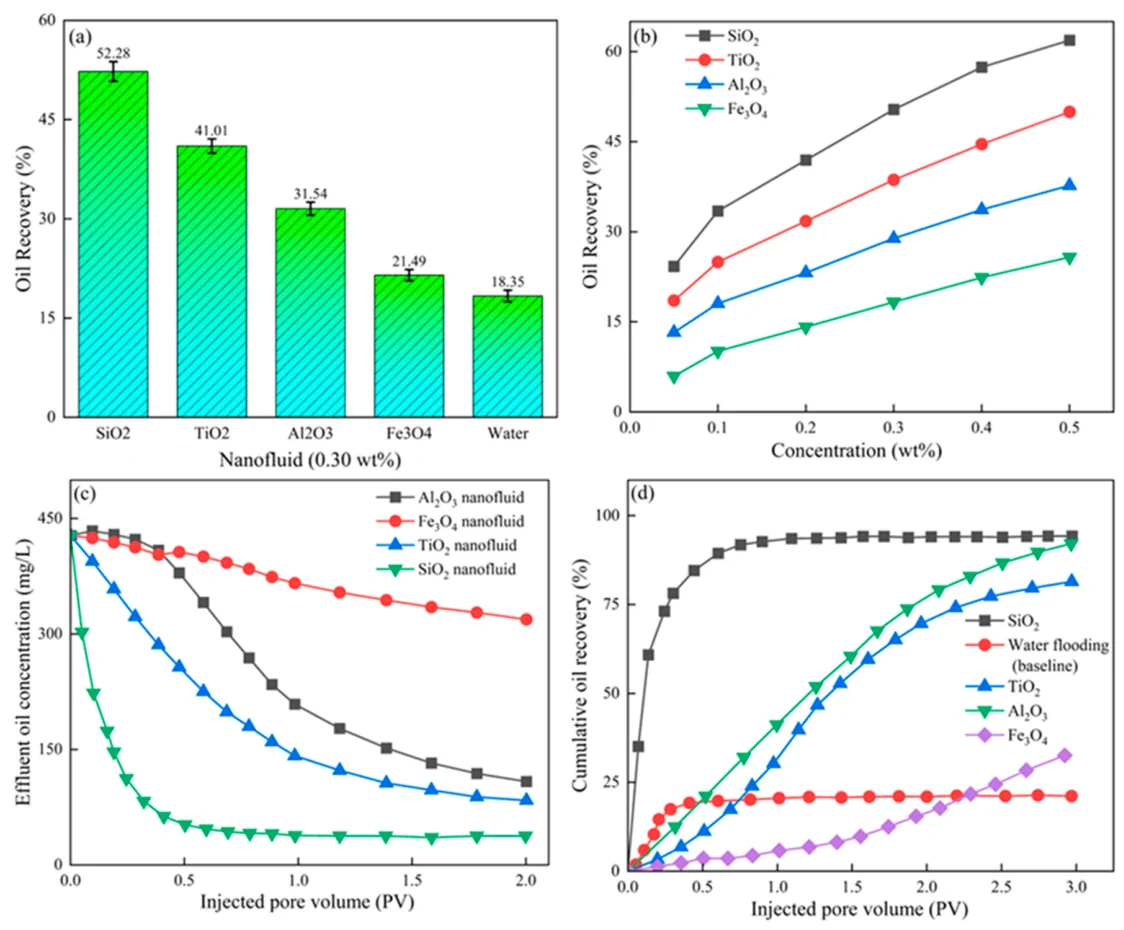
Analysis of oil displacement performance and dynamic displacement processes of different metal oxide nanofluids. (**a**) Comparison of final oil recovery rates under different nanofluids (0.30 wt%); (**b**) the effect of nanoparticle concentration on oil recovery rate; (**c**) changes in oil concentration in the product liquid during the injection of different nanofluids; (**d**) the variation pattern of cumulative oil recovery rates with the volume of injected pores in different replacement systems. Error bars represent the standard deviation of the independent replicate measurements.

As shown in Figure 8a, at the same nanoparticle concentration of 0.30 wt%, the final recovery rate of the four nanofluids is higher than that of water. Among them, the final recovery rate of SiO_2_ is 52.28%, higher than the 41.01% for TiO_2_, 31.54% for Al_2_O_3_, 21.49% for Fe_3_O_4_, and 18.35% for water flooding. The high recovery rate of SiO_2_ is not attributed to a single material property, but is the result of the joint effect of its colloidal stability, interfacial regulation, adsorption/retention behavior, and pore-scale transport properties.

First of all, SiO_2_ shows a relatively narrow hydrodynamic particle size distribution and weak aggregation tendency, which improves its accessibility to pores and pore throats, allowing more injected particles to migrate to deep sandstone areas. Secondly, the rich silanol groups on the surface of SiO_2_ can promote hydration and enhance the affinity for the aqueous phase, which is conducive to changing the wettability of the rock surface and weakening oil–rock adhesion. At the same time, SiO_2_ can also maintain effective oil–water interfacial regulation, thus further promoting the use of residual oil. More importantly, the degree of adsorption and retention of SiO_2_ is at a moderate level, rather than reaching the maximum value. This can not only maintain the interaction between the necessary particle–rock and particle–oil phases, but also reduce excessive particle loss and pore retention in the near-inlet area.

In contrast, strong adsorption capacity or surface activity itself does not necessarily lead to a higher recovery rate. For example, Fe_3_O_4_ shows strong adsorption and retention, but its more obvious aggregation tendency and pore retention limit the effective migration of particles to the deep. Therefore, the higher recovery rate of SiO_2_ is more reasonably attributed to its good comprehensive balance between dispersion stability, wetting/interfacial regulation, moderate retention, and deep transport, rather than a single performance reaching the extreme.

Figure 8b further shows that the concentration of nanoparticles is the key formulation parameter to control the oil displacement performance. As the concentration of nanoparticles increased from 0.05 wt% to 0.50 wt%, the final recovery rate of all four nanofluids increased, among which SiO_2_ showed the strongest concentration response. At lower concentrations, increasing the load of nanoparticles can increase the number of particles that can be adsorbed on the oil–water interface and the rock–fluid interface, thus enhancing the wettability alteration and interfacial regulation. This interpretation is consistent with the results of the change in contact angle and interfacial tension with concentration. However, at higher concentrations, the incremental improvement of the recovery rate gradually decreases. The increase in particle load also improves the collision frequency between particles, pore surface adsorption, and local retention. As shown in Figure 6b,c, the increase in concentration leads to a decrease in permeability and an increase in the pressure gradient, indicating that the particle–rock interaction and flow resistance in the porous medium are enhanced. Therefore, the concentration has a competitive effect: insufficient particle load will limit interface coverage and flow regulation, while excessive load may promote aggregation, retention, and injection resistance. Therefore, the appropriate concentration should be determined based on the balance between colloidal stability, interfacial regulation, pore transport, and recovery rate, not just the recovery rate.

The results of this study are generally consistent with the recent literature and further emphasize the reservoir dependence of the properties of nanoparticles. Kandiel et al. identified the type, concentration, size, and surface properties of nanoparticles as key factors in controlling wettability alteration, interfacial tension, and crude oil recovery rate, and pointed out that SiO_2_ and Al_2_O_3_ perform well under specific reservoir conditions. They also pointed out that excessive nanoparticle loads may promote aggregation and pore throat blockage. These understandings are consistent with the good performance of SiO_2_ observed in this study and the phenomenon of a decreasing increase in the recovery rate at high concentrations. However, under the unified sandstone conditions adopted by this study, the recovery rate of TiO_2_ is better than that of Al_2_O_3_, indicating that the performance sequencing of metal oxides cannot be generalized from the colloidal state to reservoir conditions. Saha and Phukan’s recent core flooding experiments also proved that nanofluids containing stable SiO_2_ can improve the wettability and residual oil recovery of heavy oil sandstone systems [38], although the formula used contains surfactants and polymers, which is different from single-component nanofluids in this study. More importantly, the results of this study further show that strong adsorption itself does not guarantee a high recovery rate: although Fe_3_O_4_ shows obvious adsorption and retention, its deep propagation is limited; in contrast, SiO_2_ achieves a more favorable balance between colloidal stability, interfacial regulation, and pore transport.

The oil concentration changes in the product liquid during different nanofluid injection processes are shown in Figure 8c. As PV injection increased, the oil concentration of the produced liquids in each system gradually decreased, indicating that residual oil was continuously used during the replacement process. Among them, SiO_2_ nanofluids declined the fastest, with a clear downward trend observed at the initial injection stage and maintaining a low level after about 1.0 PV; the TiO_2_ system followed in a downward trend; the decline in Al_2_O_3_ and Fe_3_O_4_ systems is relatively slow. Changes in oil concentration in the produced liquid reflect the influence of nanofluids on the residual oil release process. The SiO_2_ system reduces oil concentration more quickly, indicating it can act more effectively at the oil–water interface and promote crude oil desorption from the pore walls. In contrast, the Fe_3_O_4_ system maintains a relatively high oil concentration throughout the injection process, indicating relatively limited utilization efficiency for residual oil. Figure 8d further compares the variation patterns of cumulative oil recovery rates with PV injection in different replacement systems. During water flooding, cumulative oil recovery increases rapidly in the initial stage, then enters the plateau stage earlier and ultimately remains around 20%, indicating that pure water flooding easily forms a dominant flow channel, making it difficult to continue utilizing residual oil in subsequent stages. Nanofluidic displacement exhibits more sustained oil enhancement characteristics, with the SiO_2_ system rapidly increasing oil recovery rate at low PV stages, reaching over 90% after about 1.5 PV and maintaining a stable state. Although the TiO_2_ and Al_2_O_3_ systems grow at lower rates than SiO_2_, they continue to increase throughout the replacement process, ultimately significantly exceeding those of water flooding. The cumulative oil recovery rate of the Fe_3_O_4_ system has grown slowly, reaching only about 32% at 3.0 PV. These results indicate that after nanoparticles enter the pores, they can alter local flow states, delay the formation of dominant aqueous channels, and allow more crude oil to be displaced.

### 3.5. Oil–Water Separation Performance and Process Optimization

As shown in Figure S6, the oil removal behavior of four nanofluids is affected by the properties and operating conditions of nanoparticles at the same time. In Figure S5a, SiO_2_ maintained relatively stable and high oil removal efficiency within the initial oil concentration range studied, while TiO_2_, Al_2_O_3_, and Fe_3_O_4_ showed more obvious concentration-dependent changes. These differences can be understood from the competition between oil droplet–particle contact enhancement and surface site saturation and aggregation under high oil load.

At low initial oil concentrations, the number of oil droplets that can interact with the surface of nanoparticles is limited, so the adsorption and surface-active sites on the surface of the particles have not been fully utilized. As the oil concentration increases, the probability of collision and contact between oil droplets and nanoparticles increases, so the oil removal efficiency of TiO_2_ and Al_2_O_3_ in the medium concentration range is improved. However, when the oil concentration continues to rise and the amount of nanoparticles remains unchanged, the oil/particle ratio increases, and the usable sites on the surface of the particles gradually tend to be saturated. At the same time, the effects of oil droplet aggregation, particle–oil aggregate formation, and mass transfer restriction may further reduce the amount of oil that can be effectively removed, thus reducing the oil removal efficiency.

The small change in oil removal efficiency of SiO_2_ may be related to its good dispersion stability, small effective aggregation size, and silanol-rich hydrated surface, which are conducive to maintaining a large effective interface contact area in a wide range of oil concentrations. In contrast, Fe_3_O_4_ has a more obvious aggregation tendency and a larger hydrodynamic particle size. Therefore, under higher oil load, particle aggregation and particle–oil phase binding are more likely to reduce its usable surface area, resulting in a more obvious decrease in oil removal efficiency.

Therefore, the oil removal efficiency of different nanoparticles depends not only on their intrinsic adsorption ability, but also on the comprehensive balance between the effective surface area, particle dispersion state, oil/particle ratio, and oil droplet–particle interface contact.

The oil–water separation performance of nanofluids is significantly affected by the solution environment, especially pH, temperature, and salinity, because these factors change the surface charge, dispersion state, and interfacial interaction of nanoparticles. As shown in Figure S7a, as the pH changes from acidic to moderately alkaline, the oil-removal efficiency generally tends to rise and then decreases at the highest pH. This pH-dependent behavior can be understood from the acid–base charging on the surface of the oxide and the ionization of polar components in crude oil. Hydroxyl groups on the surface of metal oxides can be protonated and deprotonated in the following ways:

$$\equiv MOH+H^{+}⇌\equiv {MOH}_{2}^{+}$$

$$\equiv MOH⇌\equiv {MO}^{-}+H^{+}$$

Among them, ≡MOH represents the hydroxylated surface site. At low pH, the protonation of the oxide surface and the weak ionization of acidic polar groups in crude oil may reduce the stability of the colloid and limit the accessible surface area of nanoparticles. As the pH increases, the surface hydroxyl group gradually deprotonates, which usually increases the surface charge and improves the dispersion stability of the stable oxide system, thus increasing the effective interfacial area and the probability of nanoparticle contact with oil droplets. If there are acidic polar groups in crude oil, they can also be dissociated in the following way:

(1) $$RCOOH⇌{RCOO}^{-}+H^{+}$$

At moderately alkaline pH, the improvement of nanoparticle dispersion, the enhancement of interfacial accessibility, hydrogen-bonding/acid–base interactions, and the increase in collision frequency are conducive to the bonding of nanoparticles and oil together, thereby improving the oil-removal efficiency. However, under strong alkaline conditions, extensive deprotonation may make the surface of nanoparticles and the ionized oil droplet interface have a strong negative charge. The resulting electrostatic repulsion, combined with the enhancement of surface hydration, will weaken the binding of nanoparticles and oil phases, thus explaining the decrease in oil-removal efficiency observed at the highest pH. There are differences in this response amplitude between SiO_2_, TiO_2_, Al_2_O_3_, and Fe_3_O_4_ because their surface hydroxyl chemistry, charge behavior, hydration, and aggregation tendencies are different. In particular, the high oil-removal efficiency of SiO_2_ is consistent with its better dispersion stability and hydrated surface rich in silanol, while the low oil-removal performance of Fe_3_O_4_ is consistent with its strong aggregation tendency. Therefore, the changing trend in Figure S7a should be understood as the common result of pH-dependent surface charge, colloidal stability, and nanoparticle–oil interfacial interaction, not just the embodiment of adsorption affinity.

Figure S7b–d further show that temperature, dispersion pH, and Na_2_SO_4_ concentration also affect the oil-removal performance in a material-dependent way. These environmental variables can change the aggregation, surface charge, hydration, and interfacial accessibility of nanoparticles, so the oil-removal response generally reflects the coupling between the type of nanoparticles and the solution conditions.

As shown in Figure S8, appropriately extending the ultrasonic time can reduce the size of oil droplets, improve particle dispersion, and promote improved oil–water separation efficiency. Among them, SiO_2_ nanofluids demonstrate good overall performance under ultrasonic conditions, effectively reducing oil droplet size while maintaining high system stability; TiO_2_ and Al_2_O_3_ systems have certain improvement effects, while Fe_3_O_4_ systems are affected by particle aggregation, resulting in relatively limited ultrasonic enhancement effects. Therefore, in the preparation of nanofluids, reasonably controlling the ultrasonic time plays a direct role in obtaining a stable dispersed system and improving oil removal efficiency.

It should be noted that the oil–water separation experiment in this study adopts a laboratory simulation of oil-containing water prepared from the actual crude oil in the Dagang Oilfield, rather than the oilfield water collected on the spot. The purpose of adopting this controlled system is to exclude uncontrolled fluctuations in the chemical composition of water as much as possible, so as to independently compare the influence of nanoparticle types and operating parameters. The actual oilfield water may contain high concentrations of dissolved salts, suspended mineral particles, natural surfactants, dissolved organic matter, and other oilfield chemicals. These components may affect the aggregation, surface charge, interfacial adsorption, and oil removal properties of nanoparticles. Therefore, although the results of this study show that SiO_2_ has good oil removal potential under controlled conditions, it still needs to be further verified with representative on-site produced water before practical application.

### 3.6. Long-Term Stability and Mechanism Analysis

**Figure 9:**
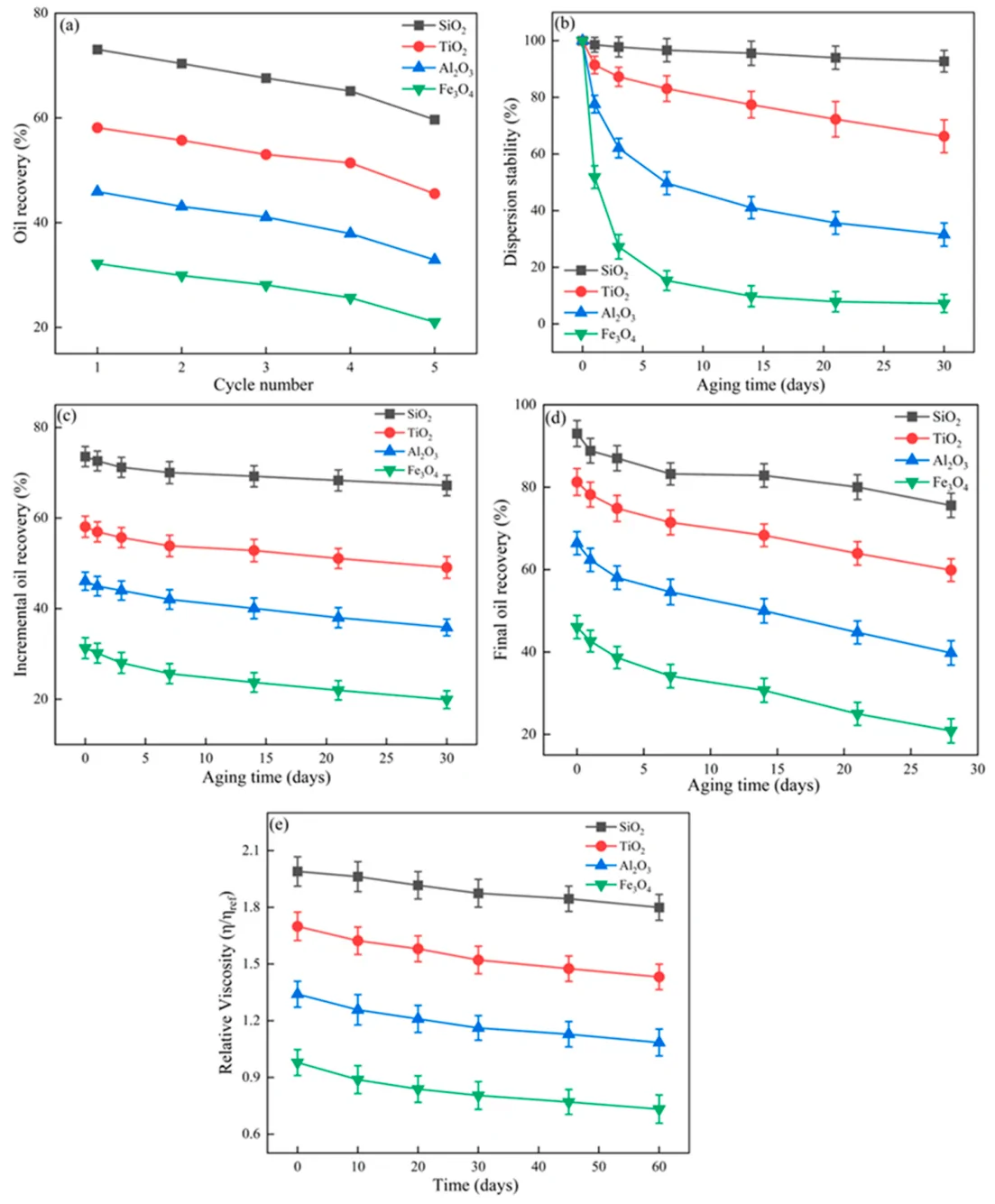
Performance changes in different metal oxide nanofluids during long-term storage and reuse. (**a**) The effect of the number of cycles on oil removal efficiency; (**b**) the effect of aging time on the dispersion stability of nanofluids; (**c**) the effect of aging time on incremental oil recovery rates; (**d**) the effect of aging time on the final oil recovery rate; (**e**) the effect of aging time on relative viscosity. Error bars represent the standard deviation of the independent replicate measurements.

As shown in Figure 9a, as the number of cycles increases from 1 to 5, the degreasing efficiency of different nanofluids gradually decreases, but the degree of decrease varies significantly. The initial degreasing efficiency of SiO_2_ nanofluids is about 72%, and after five cycles, it remains at about 60%; TiO_2_ systems reduced from about 58% to 45%; Al_2_O_3_ systems reduced from about 46% to 33%; the Fe_3_O_4_ system showed the most significant decline, dropping from about 32% to 21%. During the cycle, nanoparticles continuously participate in oil droplet adsorption and interfacial regulation. Some particle surface active sites may be covered by oil components, and repeated recycling processes may also cause irreversible accumulation, reducing their effective function. The SiO_2_ system’s performance degradation is minimal, indicating that its particle surface structure is relatively stable and can maintain good dispersion during cycling. The effect of aging time on the dispersion stability of nanofluids is shown in Figure 9b. As storage time increases from 0 days to 30 days, different systems exhibit distinct stability changes. SiO_2_ nanofluids maintained over 90% dispersion stability after 30 days, with minimal variation; TiO_2_ systems reduced their content from nearly 95% to about 65%; Al_2_O_3_ systems dropped to about 30%; the Fe_3_O_4_ system declined the fastest, maintaining only a low level after 30 days. During long-term storage, van der Waals interactions and magnetism between particles may promote agglomeration formation, while the surface hydration layer and charge repulsion can suppress particle approach. The hydroxyl structure on the surface of SiO_2_ particles enhances hydration, maintaining good spatial stability between particles, thus exhibiting high long-term stability. Figure 9c,d show the effects of aging time on incremental and final oil recovery rates, respectively. As aging time increases, the oil displacement performance of all nanofluids declines, with the SiO_2_ system showing the smallest change. The incremental oil recovery rate dropped from about 72% initially to about 68% at 30 days, and finally from about 92% to around 75%; TiO_2_ systems correspond to about 49% and 60% reduction; Al_2_O_3_ systems are reduced to about 36% and 40%, respectively; the Fe_3_O_4_ system has declined most significantly, with the final oil recovery rate remaining at only about 20%. The degradation of oil displacement performance is consistent with the long-term stability changes in nanofluids. During aging, after some particles agglomerate, their effective specific surface area decreases, and their ability to enter tiny pores and oil–water interfaces weakens, making it difficult to continuously exert wetting changes and interface regulation effects. The SiO_2_ system can maintain high oil displacement performance, mainly due to its good dispersion retention and stable interfacial effects. Figure 9e further analyzes the effect of aging time on the relative viscosity of nanofluids. Over time, the relative viscosity of all systems gradually decreases. Among them, SiO_2_ systems decreased from about 2.0 to about 1.8; TiO_2_ systems decreased from about 1.7 to 1.4; Al_2_O_3_ systems decreased from about 1.35 to 1.1; the Fe_3_O_4_ system dropped to about 0.75. The decrease in viscosity indicates that the microstructure of the fluid formed by nanoparticles gradually weakens during long-term storage, and the effective interactions between particles decrease. For intensified oil extraction processes, viscosity reduction reduces flow control ability, so long-term viscosity retention is also an important aspect of evaluating nanofluidic performance.

As shown in Figure S9, ζ potential reflects the surface charge characteristics of nanoparticles and the electrostatic repulsion between particles, serving as a key parameter for evaluating the stability of nanofluid dispersion. Salinity, temperature, and long-term storage processes in the reservoir environment all alter the charge distribution on the particle surface, thereby affecting particle aggregation behavior. Therefore, the potential variation patterns of SiO_2_, TiO_2_, Al_2_O_3_, and Fe_3_O_4_ nanofluids under different environmental conditions were analyzed. Potential changes under different environmental conditions ζ results show that the surface charge state of nanoparticles is jointly influenced by the solution ion environment, temperature, and storage time. Among them, SiO_2_ nanofluids can maintain relatively stable negative charge states under changes in salinity, temperature, and aging conditions, demonstrating good colloidal stability; the TiO_2_ and Al_2_O_3_ systems respond significantly to changes in the external environment, with stability dependent on specific conditions; due to strong interactions between magnetic particles, the Fe_3_O_4_ system has relatively limited charge regulation capability. ζ potential variation patterns correspond well with particle size, dispersion stability, and oil displacement performance, indicating that charge regulation on the surface of particles is an important factor affecting the long-term stable operation of nanofluids.

As shown in Figure S10, after nanofluids enter porous media, their particle migration, pore retention, and interactions with the rock surface directly affect fluid seepage resistance and the surge efficiency of displacement processes. Therefore, further analysis was conducted on the effects of different types of nanoparticles on the flow characteristics of porous media. From the above results, it can be seen that different nanoparticles exhibit significant differences in migration characteristics in porous media due to differences in particle size, surface properties, and interaction modes with the rock surface. SiO_2_ nanofluids exhibit strong resistance regulation capabilities, while Fe_3_O_4_ nanofluids can produce a pronounced resistance response while maintaining low pressure loss, indicating that different types of nanomaterials have different mechanisms for improving flow ratio and regulating pore flow. These results provide an experimental basis for analyzing the effects of enhanced oil extraction by nanofluids by combining interface properties and oil displacement efficiency.

**Figure 10:**
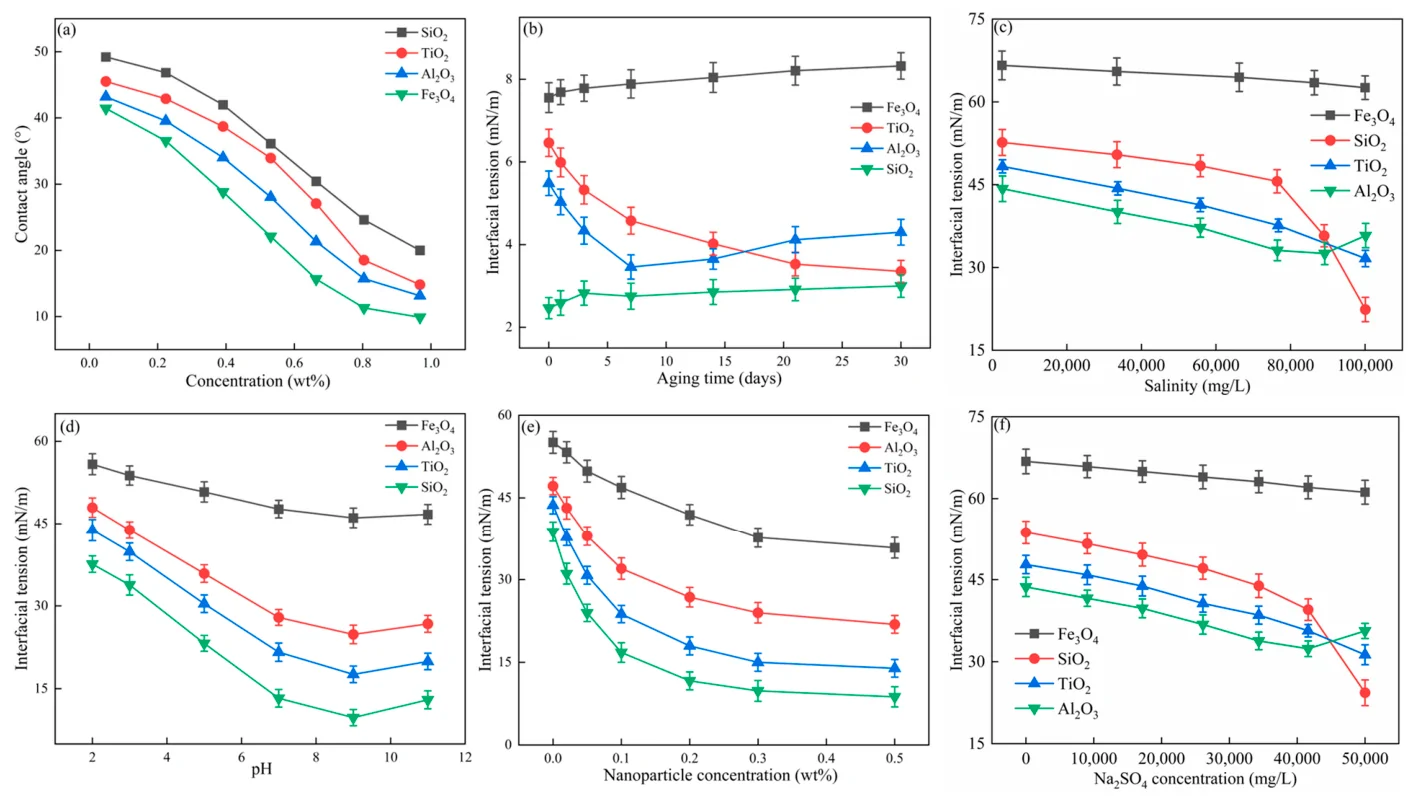
Effects of different metal oxide nanofluids on the properties of the oil–water interface. (**a**) The effect of nanoparticle concentration on the angle of contact on the rock surface; (**b**) the effect of aging time on the tension of the oil–water interface; (**c**) the effect of salinity on interfacial tension; (**d**) the effect of pH on interfacial tension; (**e**) the effect of nanoparticle concentration on interfacial tension; (**f**) the effect of sodium sulfate concentration on interfacial tension. Error bars represent the standard deviation of the independent replicate measurements.

As shown in Figure 10a, as the concentration of nanoparticles increases, the contact angles after treatment with all four nanofluids gradually decrease, indicating that nanoparticles can promote the transition of rock surfaces from relatively oleophilic to hydrophilic. Among them, the contact angle for SiO_2_ nanofluids is reduced from about 49° to 20°, and for TiO_2_ systems, from 45° to about 15°, and for the Al_2_O_3_ and Fe_3_O_4_ systems, it is reduced to about 13° and 10°, respectively. The reduction in contact angle indicates that nanoparticles can adsorb on solid surfaces and alter the interfacial equilibrium between oil, water, and rock. The differences between different materials are related to the chemical properties of the particle surface. The hydroxyl groups on the oxide surface can enhance the interaction between the aqueous phase and the rock surface, weakening the adhesion between crude oil and mineral surfaces. Figure 10b examines the effect of aging time on interfacial tension during nanofluid storage. As the aging time increases from 0 days to 30 days, different nanofluids exhibit varying degrees of interfacial tension changes. Among them, the interfacial tension of Fe_3_O_4_ nanofluids has consistently remained high, increasing from about 7.5 mN/m to 8.3 mN/m; SiO_2_ systems remain in a relatively low range, around 2.5~3.0 mN/m. The TiO_2_ and Al_2_O_3_ systems also remain relatively stable during aging. These results indicate that when nanoparticles remain in the aqueous phase for a long time, the stability of their surface structure affects their interfacial activity. The SiO_2_ system can maintain low interfacial tension for a long time, indicating stable particle dispersion and continuous participation in oil–water interface regulation. The effect of salinity changes on interfacial tension is shown in Figure 10c. As salinity increases from 0 to 100,000 mg/L, the interfacial tension of different nanofluids changes, but the trends differ significantly. TiO_2_ nanofluids show the most pronounced downward trend, dropping from about 52 mN/m to 20 mN/m; SiO_2_ systems decrease from about 65 mN/m to 33 mN/m; the Al_2_O_3_ system decreases to about 35 mN/m, while the Fe_3_O_4_ system shows less change. As salinity increases, the ionic strength of the solution increases, altering the charge distribution on the nanoparticle surface and the adsorption state at the oil–water interface. On one hand, ions can promote the migration of some particles toward the interface region; on the other hand, excessively high ion concentration compresses the double electric layer on the particle surface, reducing particle stability. Therefore, different nanomaterials respond differently to salinity changes due to their varying surface charge properties. The effect of pH on interfacial tension is shown in Figure 10d. As the pH increases from 2 to 11, the interfacial tension of the four nanofluids generally shows a downward trend. Among them, the SiO_2_ system showed the most significant change, dropping from about 40 mN/m to 10 mN/m; the TiO_2_ and Al_2_O_3_ systems are reduced to about 26 mN/m and 20 mN/m, respectively; the decline in the Fe_3_O_4_ system is small. pH changes affect the form of hydroxyl groups on the surface of nanoparticles. Under alkaline conditions, the hydroxyl groups on the surface undergo deprotonation, increasing the negative charge on the particle surface, which facilitates dispersion of particles in the aqueous phase and the rearrangement of the oil–water interface. Therefore, suitable pH conditions can enhance the interface regulation ability of nanoparticles. The effect of nanoparticle concentration on interfacial tension is shown in Figure 10e. As the concentration increases from 0 to 0.5 wt%, interfacial tension in all systems gradually decreases, with SiO_2_ nanofluids showing the largest decrease, from about 58 mN/m to 8 mN/m; the TiO_2_, Al_2_O_3_, and Fe_3_O_4_ systems reduce to approximately 22, 14, and 35 mN/m, respectively. As the concentration of nanoparticles increases, more particles can migrate and adsorb at the oil–water interface, forming a protective layer and thereby reducing interfacial free energy. As the concentration increases further, the decrease in interfacial tension tends to slow down, indicating that interfacial adsorption is gradually reaching equilibrium. Figure 10f further analyzes the effect of Na_2_SO_4_ concentration on interfacial tension. As sodium sulfate concentration increases, interfacial tension in all systems shows a downward trend, with the most significant change in TiO_2_ nanofluids, dropping to about 25 mN/m at 50,000 mg/L; the SiO_2_ system reduced to about 37 mN/m; the decline in Al_2_O_3_ and Fe_3_O_4_ systems is relatively small. The introduction of sulfate ions alters the solution ion environment and affects the charge state and interfacial adsorption behavior of nanoparticles, causing adjustments in the oil–water interface structure.

From the perspective of modeling, the transport problem of nanoparticles in reservoir porous media can also be described by the fractional-order method. Traditional transport models generally assume that the process has local and Markov properties, but when nanoparticles migrate in heterogeneous porous media, they are often accompanied by aggregation, adsorption–desorption, pore throat retention, and delayed transport. These processes may cause the response to be non-local and memory-dependent. Martínez-Ayala et al. have proved that Caputo fractional calculus can effectively depict the dynamic behavior with memory-dependent characteristics in complex aqueous systems. Although their work is mainly aimed at laser-induced thermal behavior and pollutant detection, and does not involve nanoparticle transport, it at least shows that fractional-order models can capture processes that deviate from classical local descriptions. More direct evidence comes from the research of Pan et al., who established a spatial fractional transport model for nanofluids in disordered porous media, and linked the fractional form with the anomalous migration behavior of nanoparticles [36]. Based on these works, future nanofluid EOR research can combine the fractional advection-diffusion model with the experimentally obtained adsorption, aggregation, and retention parameters, so as to quantitatively describe the propagation process of nanoparticles in heterogeneous pore networks, and establish a connection between colloidal-scale transport and macroscopic recovery properties.

## 4. Conclusions

Under unified experimental conditions, the nanofluids SiO_2_, TiO_2_, Al_2_O_3_, and Fe_3_O_4_ exhibit significantly different structure-performance relationships. SiO_2_ has a narrow hydrodynamic particle size distribution in the aqueous phase, with a polydispersity index of about 0.15 under neutral conditions, and charge decay caused by salinity, temperature, and aging is relatively limited; Fe_3_O_4_ is affected by interactions between magnetic particles, with agglomeration and long-term sedimentation becoming more pronounced. The hydroxyl groups and hydrated layers on the particle surface not only affect colloid stability but also determine the degree of disturbance particles have to the continuous phase structure. At 0.5 wt%, SiO_2_ has dynamic viscosities and relative viscosities of about 3.6 mPa·s and 4.5, respectively, and exhibits strong shear-thinning characteristics; TiO_2_ is followed by Al_2_O_3_ and Fe_3_ The degree of viscosity increase in O_4_ is small.

There is no simple positive correlation between adsorption capacity and oil displacement performance. Fe_3_O_4_ adsorption of crude oil components and rock surfaces is relatively high, with significant dynamic adsorption loss and residual resistance, but strong adsorption, magnetic agglomeration, and pore throat retention shorten its effective migration distance. SiO_2_ has a relatively low static adsorption capacity but maintains good dispersion and propagation during flow, continuously altering the oil–water–rock interface. The resulting improvements in wettability, reduced interfacial tension, continuous phase viscosity, and local flow path redistribution all promote the use of residual oil. These results indicate that moderate adsorption is more conducive to nanoparticles exerting their effect deep into the reservoir than simply pursuing maximum adsorption capacity.

Core displacement results are consistent with the above findings. Under 0.30 wt% conditions, the final oil recovery rate of SiO_2_ nanofluids was 52.28%, including TiO_2_, Al_2_O_3_, and Fe_3_O_4_, and water flooding was 41.01%, 31.54%, 21.49%, and 18.35%, respectively. At a SiO_2_ concentration of 0.2 wt%, the incremental oil recovery rate is about 36%; after the injection section plug increases to 2.0 times the pore volume, the oil recovery rate is about 92%. Weakly alkaline environments, moderate injection rates, and moderate core permeability are more conducive to balancing particle residence time and deep propagation, while excessively high concentrations, large particle sizes, or excessively fast flow velocities weaken interfacial effects and even increase the risk of local blockage. The comprehensive oil displacement performance of the four materials is, in order: SiO_2_, TiO_2_, Al_2_O_3_, and Fe_3_O_4_, but this ranking comes from the superposition of multiple properties and cannot be directly derived from a single parameter.

Oil–water separation and long-term aging experiments further verify the importance of the material’s dispersed state. The SiO_2_ system achieves a degreasing efficiency of 92~94% under suitable particle concentration, contact time, and ultrasonic conditions, with a stability index close to 95% after about 20 min of ultrasonic treatment; after 30 days of aging, its dispersion stability remains above 90%, and after five cycles, it retains about 60% degreasing efficiency. TiO_2_ has good mid-term stability, while Al_2_O_3_ and Fe_3_O_4_ are more prone to performance loss due to agglomeration and surface activity decay. Therefore, it can be concluded that material screening for nanofluids should simultaneously assess surfactancy, viscosity capacity, adsorption loss, pressure response, and long-term stability. For the four oxides examined in this study, the advantages of SiO_2_ do not come from any particular extreme performance but from a coordinated combination of stable dispersion, moderate adsorption, interfacial regulation, and pore propagation. Follow-up research should further verify the long-term injection behavior of these nanofluids under actual formation water, heterogeneous rock cores, and high temperature and high pressure conditions. In addition, representative on-site produced water composed of actual salinity, suspended solids, dissolved organic matter, and residual oilfield chemicals should be used to further evaluate the oil removal performance of SiO_2_, so as to evaluate its application potential in actual oil–water separation. At the same time, its engineering application boundaries should also be evaluated in combination with nanoparticle recovery, reservoir damage, and economic feasibility. In addition, future work should combine SEM or TEM characterization with controlled particle-morphology experiments to distinguish the effects of particle shape, surface structure, aggregation state, and composition. These characterization results could then be incorporated into fractional-order transport models to more quantitatively describe nanoparticle aggregation, retention, and anomalous transport in heterogeneous pore networks.

## Data Availability

The data presented in this study are available on request from the corresponding authors.

## Supplementary Materials

The following supporting information can be downloaded at: https://www.mdpi.com/article/10.3390/ma19194199/s1, Table S1: Main Nanoparticles and Chemical Reagents; Figure S1: Schematic illustration of the main experimental systems used in this study. Figure S2: Viscosity changes and empirical model analysis of different metal oxide nanofluids. Figure S3: The effects of different factors on the adsorption behavior and flow regulation ability of metal oxide nanofluids. Figure S4: Effects of different experimental parameters on the oil displacement performance of metal oxide nanofluids. Figure S5: Effects of different reservoir environmental parameters on the oil displacement performance of metal oxide nanofluids. Figure S6: Effects of different experimental conditions on the oil-water separation efficiency of metal oxide nanofluids. Figure S7: Effects of different operating conditions on the degreasing performance of metal oxide nanofluids. Figure S8: Effect of ultrasonic treatment time on the dispersion behavior and degreasing performance of oil droplets in different metal oxide nanofluids. Figure S9: Effects of different environmental factors on the ζ potential of metal oxide nanofluids. Figure S10: The osmotic flow of different metal oxide nanofluids in porous media varies.
