# Supplementary material for: Structure–Performance Relationships of Metal Oxide Nanofluids for Enhanced Oil Recovery: Balancing Colloidal Stability, Interfacial Regulation, and Pore Transport
Source: Materials (Basel). 2026 Sep 30;19(19):4199. doi: 10.3390/ma19194199 (PMC13666293; doi:10.3390/ma19194199)
Supplement: Supplementary file 1 [file materials-19-04199-s001.zip › materials-4508608-supplementary.pdf]

S1. Materials and Detailed Nanofluid Preparation

S1.1. Materials, Crude-Oil Pretreatment, and Core Preparation

SiO_2, TiO_2, Al_2O_3 and Fe_3O_4 nanoparticles are used as four representative metal oxide materials. Their nominal primary particle sizes are 20, 25, 30 and 20 nm respectively, and the nominal purity is not less than 99%. Sodium chloride (NaCl), anhydrous sodium sulphate (Na_2SO_4), potassium chloride (KCl), hydrochloric acid (HCl), sodium hydroxide (NaOH), anhydrous ethanol, toluene and n-hexane are all analytically pure. All aqueous solutions are prepared with deionised water with a resistivity of 18.2 MΩ·cm.

Crude oil is taken from the Dagang Oilfield in Tianjin, China. Before use, leave the crude oil at 60 °C for 2 hours, and then centrifuge at 3000 rpm for 20 minutes to remove free water and suspended mechanical impurities. Unless otherwise stated, all displacement experiments use the same batch of crude oil to minimise the impact of differences in crude oil composition on interfacial properties and recovery rates.

Natural sandstone cores are used in porous medium experiments and rock core displacement experiments. The diameter of the cylindrical rock core is 25.0 ± 0.1 mm and the length is 50.0 ± 0.5 mm. Except for the permeability adaptability experiment, the gas measurement permeability of the reference rock core is controlled at 500 ± 25 mD; the permeability range of the rock core used in the permeability adaptability experiment is about 50–1500 mD. Before use, the core is extracted with toluene and ethanol in turn, and extraction with each solvent is performed for 24 h until the outflow liquid has no obvious colour. The cleaned rock core is dried at 105 °C for 24 h, and then stored in the desiccator for later use. The above content is taken directly from the latest material part.

Table S1. Main Nanoparticles and Chemical Reagents

Materials	Chemical formula or CAS number	Specifications
Silicon dioxide nanoparticles	SiO_2，7631-86-9	Nominal particle size 20 nm, purity ≥99%
Titanium dioxide nanoparticles	TiO_2，13463-67-7	Nominal particle size 25 nm, purity ≥ 99%
Alumina nanoparticles	Al_2O_3，1344-28-1	Nominal particle size 30 nm, purity ≥ 99%
Ferric tetroxide nanoparticles	Fe_3O_4，1317-61-9	Nominal particle size 20 nm, purity ≥99%
Sodium chloride	NaCl，7647-14-5	analytical pure
Anhydrous sodium sulfate	Na_2SO_4，7757-82-6	analytical pure
Hydrochloric acid	HCl，7647-01-0	analytical pure
Sodium hydroxide	NaOH，1310-73-2	analytical pure
n-Hexane	C_6H_{14}，110-54-3	analytical pure
Toluene	C_7H_8，108-88-3	analytical pure
Anhydrous ethanol	C_2H_5OH，64-17-5	analytical pure

S1.2. Nanofluid Preparation

Before use, the nanoparticles are vacuum-dried at 105 ° C for 12 hours to minimise the impact of physically adsorbed water on weighing and rheological measurement. According to

the target mass concentration, the predetermined mass of nanoparticles is slowly added to the deionised water or pre-prepared electrolyte solution.

The mass concentration of nanoparticles is calculated as follows:

$$C_{\mathrm{NP}} = \frac{m_{\mathrm{NP}}}{m_{\mathrm{NP}} + m_l} \times 100\% \qquad \text{(S1)}$$

where C_{NP} is the nanoparticle mass concentration (wt%), m_{NP} is the mass of nanoparticles (g), and m_l is the mass of the liquid phase (g).

After adding nanoparticles, the dispersion is first stirred magnetically at 1000 rpm for 30 minutes to promote preliminary wetting and dispersion. Then, the probe-type ultrasonic processor is further used for dispersion, with a frequency of 20 kHz, a rated power of 500 W, a working amplitude of 40%, and a pulse mode of 5 s on and 3 s off, lasting 60 min. During the ultrasonic process, the sample container is placed in an ice water bath and the temperature is maintained at 25 ± 2 °C to reduce the effect of excessive heating on the surface state of nanoparticles.

Except for the ultrasonic time experiment, all nanofluids are prepared according to the same steps. Colloidal, rheological and interfacial measurements are usually carried out within 30 minutes after the ultrasound. If the sample with high concentration or high salinity has obvious sedimentation, only gently invert and mix before measurement, and no additional ultrasonic treatment should be used to avoid artificially restoring the original dispersion state.

S1.3. Concentration, pH, Salinity, and Particle-Size Conditions

The concentration dependence experiment is carried out in the range of 0.01 − 0.50 wt%, and the representative concentration points are 0.01, 0.02, 0.05, 0.10, 0.20, 0.30, 0.40 and 0.50 wt%, which are selected according to the measured properties. In order to directly compare the four metal oxides, 0.30 wt% is used as the reference concentration; the concentration optimisation is evaluated separately in the range of 0 − 0.50 wt%.

Unless otherwise stated, the benchmark pH is adjusted to 8.0 ± 0.1 using 0.10 mol/L HCl or 0.10 mol/L NaOH. After adjustment, continue to stir for 10 min and re-measure the pH, and repeat the operation until the pH change is less than 0.05.

The electrolyte effect was investigated by NaCl and Na_2SO_4. The mass fraction of NaCl is set to 0, 0.5, 1.0, 3.0, 5.0 and 7.0 wt% respectively; the concentration of Na_2SO_4 is set to 0, 1.0 × 10^4, 2.0 × 10^4, 3.0 × 10^4, 4.0 × 10^4 and 5.0 × 10^4 mg/L, the total salinity of the relevant experiments is up to about 1.0 × 10^5 mg/L. The salt solution is completely dissolved before adding nanoparticles to reduce the irreversible aggregation caused by local high ionic strength.

The particle size effect experiment is based on the hydrodynamic particle size determined by DLS, not the primary particle size reported by the supplier. By changing the ultrasonic energy input and graded centrifugation conditions, a sample with a hydrodynamic particle size of about 50 − 1000 nm is obtained. Re-measure the peak hydrodynamic particle size and PDI of each group before use, and only select samples with a deviation of less than 10% from the target particle size.

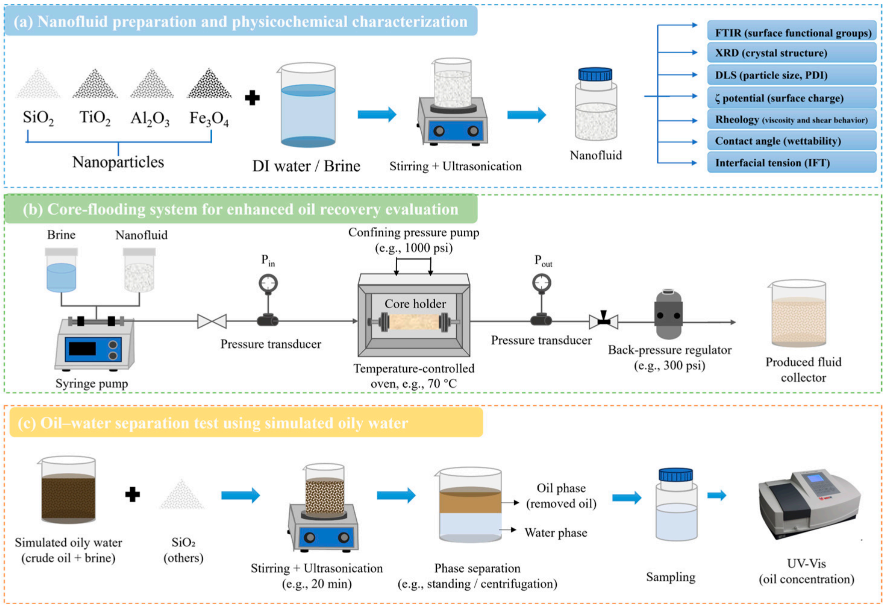

Figure S1. Schematic illustration of the main experimental systems used in this study: (a) nanofluid preparation and physicochemical characterization, (b) core-flooding system for enhanced oil recovery evaluation, and (c) oil–water separation test using simulated oily water.

S2. Physicochemical Characterization

S2.1. Fourier Transform Infrared Spectroscopy and X-ray Diffraction

The surface functional group is characterised by Fourier transform infrared spectroscopy. The dried sample was analysed in attenuated total reflection mode, with a wavenumber range of 4000–400 cm^{-1} and a resolution of 4 cm^{-1}, and 16 scans were collected for each sample. Collect the air background before each measurement.

The crystal structure of nanoparticles is characterised by X-ray diffraction, using Cu Kα radiation (λ =0.15406 nm), and the working voltage and current are 40 kV and 40 mA respectively. The scanning range is 5 – 80° , the step size is 0.02° , and the scanning rate is 2° min^{-1}. The obtained diffraction pattern is compared with the standard PDF reference card. All nanoparticle samples were dried at 105 °C for 12 h before characterisation.

S2.2. Surface Acid–Base Response and Apparent Point of Zero Charge

The pH drift method was used to evaluate the surface acid–base response of nanoparticles with reference to the existing method of oxide surface charging [41]. A 20 mL aqueous solution series with initial pH values of 2, 3, 4, 5, 6, 7, 8, 9, 10, and 11, respectively, was prepared using 0.10 mol L^{-1} HCl or NaOH. Then 0.10 g of nanoparticles was added to each solution, and the suspension was oscillated at 150 r/min and 25 °C for 24 h so that the surface acid–base equilibrium could be established.

After equilibration, the suspension was centrifuged at 8000 r/min for 10 min, and the final pH of the supernatant was determined. The pH change was calculated by the following formula:

$$\Delta pH = pH_f - pH_i \qquad (S2)$$

where pH_i and pH_f are the initial and final equilibrium pH values, respectively. The apparent point of zero charge (apparent pH_{PZC}) was identified as the initial pH at which

$$\Delta pH = 0 \qquad (S3)$$

that is, the condition when the final pH was equal to the initial pH. The term "apparent" is used here because the zero-charge condition obtained by the pH drift method may be affected by experimental factors such as solid-to-liquid ratio, ionic strength, and solution composition [41]. The pH meter was calibrated daily with standard buffer solutions of pH 4.00, 7.00, and 10.01. Each condition was tested independently three times.

S2.3. Hydrodynamic Particle Size, PDI, and ζ Potential

Using dynamic light scattering and electrophoretic light scattering, Z-average hydrodynamic particle size, polydispersity index (PDI) and ζ potential were determined by referring to the commonly used operating procedures in nanofluid characterization [40]. The instrument is equipped with a 633 nm helium-neon laser. Disposable polystyrene cuvettes are used for particle size measurement, and folded capillary electrode cells are used for ζ potential measurement.

The sample is diluted with a base solution having the same pH and salinity as the corresponding original sample, and a consistent dilution ratio is adopted in each experimental group. After loading, each sample is equilibrated at 25 ° C for 2 min. The particle size is measured in 173 ° backscattering mode. The relationship between the hydrodynamic diameter and the translational diffusion coefficient can be expressed by the Stokes − Einstein formula:

$$d_h = \frac{k_B T}{3\pi\eta D_t} \qquad (S4)$$

Where k_B, T, and η are the Boltzmann constant, absolute temperature, and dispersion medium viscosity, respectively.

And the zeta potential is calculated by electrophoretic mobility through the Smoluchowski model.

$$\zeta = \frac{\mu_e \eta}{\varepsilon_r \varepsilon_0} \qquad (S5)$$

where μ_e is the electrophoretic mobility, η is the medium viscosity, ε_r is the relative permittivity, and ε_0 is the vacuum permittivity.

The Smoluchowski approximation was used to convert electrophoretic mobility into ζ potential.

Each sample should be measured independently at least three times, and each measurement should have no fewer than 10 sub-cycles, and the results are expressed as mean ± standard deviation.

The concentration range of the experimental investigation is 0 − 0.50 wt%, the pH range is 2 − 11, and the temperature range is 25 − 85 ° C. The samples for salinity and ageing experiments are collected according to the corresponding $NaCl/Na_2SO_4$ concentration and storage time. The ageing sample is not ultrasonicated again before measurement, and only gently inverted five times to retain the actual ageing state.

S3. Rheological and Pipe-Flow Measurements

S3.1. Viscosity and Shear Behavior

The stress-controlled rotational rheometer is used to determine the rheological properties of nanofluids. The instrument is equipped with a cone-and-plate or parallel-plate measuring system and a circulating temperature control device. The temperature fluctuation during the measurement process is controlled within ± 0.1 ° C. Before measurement, use a standard-viscosity liquid and deionised water to calibrate the instrument at 25 ° C.

After the sample is added, the sample should be left for 3 min to reduce the instantaneous disturbance caused by the addition of the sample. Then pre-shear 60 s at 50 s^{-1}, then set still for 60 s, and then start the formal measurement.

The concentration-dependent viscosity was determined under the condition of 25 °C and a shear rate of 100 s^{-1}, and the concentration range was 0–0.50 wt%. The shear rate scan covers 1–1000 s^{-1} according to the logarithmic scale, and the stable viscosity is recorded after each shear rate point is maintained for at least 10 s. Temperature-dependent measurement is carried out in the range of $20-100$ ° C, and each temperature point is equilibrated for 5 min. The salinity of salinity-dependent rheological measurement is up to 1.0 × 10^5 mg/L. All viscosity measurements are repeated three times independently.

The shear-dependent response can be described using the power-law relationship:

$$\tau = K\dot{\gamma}^{n} \quad \text{(S6)}$$

$$\eta_a = \frac{\tau}{\dot{\gamma}} = K\dot{\gamma}^{n-1} \qquad (S7)$$

where τ is the shear stress, $\dot{\gamma}$ is the shear rate, K is the consistency index, and n is the flow-behavior index. When $n < 1$, the system exhibits shear-thinning behavior.

The relative viscosity is calculated as follows:

$$\eta_r = \frac{\eta_{\mathrm{nf}}}{\eta_0} \qquad \text{(S8)}$$

where $\eta_r$is the relative viscosity, $\eta_{\mathrm{nf}}$is the dynamic viscosity of the nanofluid, and $\eta_0$is the viscosity of the corresponding base liquid under the same temperature and shear conditions.

When necessary, the mass concentration of nanoparticles is converted into volume fraction by the following formula:

$$\phi = \frac{m_{\mathrm{NP}}/\rho_{\mathrm{NP}}}{m_{\mathrm{NP}}/\rho_{\mathrm{NP}} + m_l/\rho_l} \qquad \text{(S9)}$$

where $\phi$is the nanoparticle volume fraction, $\rho_{\mathrm{NP}}$is the nanoparticle density, and $\rho_l$is the density of the liquid phase.

S3.2. Reynolds Number, Friction Factor, and Pressure Gradient

The pipeline resistance evaluation adopts a stainless steel circulation system, equipped with a constant flow pump and a differential pressure sensor. The pressure sensor range is $0-500$ kPa, and the accuracy is $\pm 0.25\%$ of the full scale. Under each flow rate, the data is recorded after the pressure fluctuation is maintained within 1% and lasts for at least 5 minutes. The range of Reynolds number examined is about 5.0×10^3–1.0×10^5.

The Reynolds number is calculated as follows:

$$Re = \frac{\rho u D}{\eta} \qquad \text{(S10)}$$

where ρ is fluid density, u is the average flow velocity, D is the inner diameter of the test section, and η is the dynamic viscosity.

The Darcy friction factor was calculated as:

$$f = \frac{2\Delta PD}{\rho u^2 L} \qquad (S11)$$

where ΔP is the pressure drop across the test section and L is the effective pressure-measurement length.

The pressure gradient was calculated as:

$$G = \frac{\Delta P}{L} \qquad (S12)$$

where G is the pressure gradient.

S3.3. Empirical Viscosity Model

The concentration dependence of relative viscosity is analysed by Einstein's dilute-suspension model and empirical exponential model. For a dilute suspension, the Einstein model can be expressed as:

$$\eta_r = 1 + 2.5\phi \qquad (S13)$$

where η_r is the relative viscosity and ϕ is the particle volume fraction.

The expression of the empirical model is:

$$\eta_r = \exp(aC + bC^2) \qquad (S14)$$

where C is the nanoparticle mass concentration and a and b are fitting parameters.

Model parameters are obtained by nonlinear least squares fitting. The fitting quality is evaluated by the coefficient of determination, root-mean-square error and mean absolute relative deviation. All nanoparticle systems adopt the same fitting steps and model forms. This part corresponds to Section 2.4.3 of the manuscript.

S4. Wettability and Oil–Water Interfacial-Tension Measurements

S4.1. Rock-Surface Pretreatment and Contact-Angle Measurement

The contact angle sample is taken from the same sandstone material used in the core experiment, and the size is about 20 mm × 20 mm × 3 mm. The sample is wet-ground with 400-mesh, 800-mesh and 1200-mesh sandpaper in turn, and then polished with 0.05 μm alumina polishing solution. Then ultrasonic cleaning was performed in toluene, ethanol and deionised water for 15 min, and then the sample was dried at 60 °C for 12 h.

For oil-wet samples, the cleaned rock sample is immersed in crude oil and aged at 60 °C for 7 days. After ageing, the oil loosely attached to the surface is quickly removed using n-hexane, and then the sample is sealed for 2 hours.

For the wettability change experiment caused by nanofluids, the rock sample is soaked in the designated nanofluid for 24 hours, and the soaking conditions are consistent with the corresponding particle type, concentration, pH, salinity or temperature. After treatment, the sample is gently rinsed with the corresponding basic solution to remove the unbound particles; ultrasonic cleaning is not performed.

The contact angle is determined by an optical contact angle meter. A 3.0 μL droplet is slowly formed on the surface of the sample, and the contact angle is recorded after 60 s. At least

five different positions are measured on each sample, and the results are expressed as mean and standard deviation. For an ideal smooth and chemically homogeneous surface, the equilibrium contact angle can be related to the interfacial tensions through Young's equation:

$$\gamma_{SO} - \gamma_{SW} = \gamma_{OW} \cos\theta_w \qquad (S15)$$

where γ_{SO} is the solid–oil interfacial tension, γ_{SW} is the solid–water interfacial tension, γ_{OW} is the oil–water interfacial tension, and θ_w is the contact angle measured through the aqueous phase.

Because sandstone is rough and chemically heterogeneous, the experimentally measured value is treated as an apparent contact angle rather than an ideal Young equilibrium angle [42,43].

Clean sandstone samples are used to evaluate the impact of concentration changes on the inherent hydrophilicity of rocks, while crude-oil-aged samples are used to evaluate the wettability changes caused by pH, temperature, salinity and displacement. These two groups of samples represent different initial surface states, so they are interpreted separately.

S4.2. Oil–Water Interfacial Tension

The tension of the oil-water interface is determined by the pendant-drop method. Place the nanofluid in a transparent quartz tank, and use a stainless steel needle to generate about 8 μL of crude oil droplets in the continuous water phase.

The droplet contour is fitted by the Young-Laplace equation.

$$\Delta P = \gamma_{OW}\left(\frac{1}{R_1} + \frac{1}{R_2}\right) \qquad (S16)$$

where ΔP is the pressure difference across the oil–water interface, γ_{OW} is the oil–water interfacial tension, and R_1 and R_2 are the two principal radii of curvature.

The interfacial tension was obtained by fitting the measured droplet profile to the Young–Laplace equation [42,43].

Except for temperature-dependent experiments, measurements are carried out at 25 ± 0.1 °C. Each droplet is monitored for 30 minutes, and the stabilized value is recorded when the interfacial tension changes less than 0.1 mN/m.

Concentration, pH, total salinity, Na_2SO_4 concentration and ageing time are adjusted according to the corresponding experimental design. Analyse at least three independently prepared droplets under each condition.

S5. Adsorption Measurements

S5.1. Static and Kinetic Adsorption of Crude-Oil Components

Static adsorption is evaluated by the batch equilibrium method. Similar batch and dynamic adsorption approaches have been applied in nanoparticle-assisted EOR studies [43,44]. Prepare an oil-containing aqueous solution with an initial oil concentration of 5 − 100 mg/L, take 50 mL of oil-containing water and put it in a 100 mL conical flask with a stopper, and add 0.20 g of dried nanoparticles. The mixture is shaken for 24 hours at 25 °C and 150 rpm to approach the adsorption equilibrium.

After the adsorption, the sample is centrifuged at 10000 r/min for 10 min to determine the concentration of residual oil in the supernatant.

The adsorption kinetics experiment uses the same solid-liquid ratio, and the contact time is set to 1, 5, 10, 20, 40, 60, 90, 120, 150 and 180 min respectively.

The equilibrium adsorption capacity and time-dependent adsorption capacity are calculated by the following formula:

$$q_e = \frac{(C_0 - C_e)V}{m} \qquad \text{(S8a)}$$

$$q_t = \frac{(C_0 - C_t)V}{m} \qquad \text{(S8b)}$$

where q_e and q_t are the adsorption capacities at equilibrium and time t (mg/g), respectively; C_0, C_e, and C_t are the initial, equilibrium, and time-dependent oil concentrations (mg/L), respectively; V is the solution volume (L); and m is the nanoparticle mass (g).

The oil concentration was determined by n-hexane extraction followed by ultraviolet spectrophotometry. The water sample is mixed with n-hexane in a volume ratio of 1:1 and is shaken for 15 min. After complete phase separation, the upper n-hexane phase is collected and the absorbance is determined at 285 nm. The standard curve of crude oil/n-hexane is used for quantification, and its $R^2 \geq 0.995$.

S5.2. Nanoparticle Adsorption on Rock Surfaces

The cleaned and dried sandstone core is crushed and sieved to 40 − 60 mesh. Weigh 5.00 g of rock powder, add it to 50 mL of 0.20 wt% nanofluid, and shake the mixture at 25 ° C and 150 rpm; contact times of up to 1200 min are investigated.

After the adsorption, the suspension is centrifuged. After the supernatant is subjected to acid digestion, the concentrations of Si, Ti, Al or Fe are determined by inductively coupled plasma optical emission spectrometry, and the remaining nanoparticle concentration is calculated from the corresponding elemental concentration.

The adsorption of nanoparticles on rock per unit mass is calculated according to the same mass balance relationship as formula (S8), in which the nanoparticle concentration replaces the oil concentration and the rock mass replaces m.

S5.3. Dynamic Adsorption Loss

Dynamic adsorption is evaluated by water-saturated sandstone core, following the general dynamic-retention concept reported for nanosilica transport in porous rock systems [44]. Nanofluid is injected at a specified flow rate, and effluent samples are collected at intervals of 0.10 PV until the concentration of nanoparticles in the effluent is close to the injected concentration.

The flow rate range examined is 0.5 − 8 mL/min, the temperature range is 25 − 85 ° C, and the concentration range of nanoparticles is 0.05 − 0.50 wt%. The concentration of nanoparticles in the effluent is determined by measuring the concentration of Si, Ti, Al or Fe by inductively coupled plasma optical emission spectrometry.

The dynamic adsorption loss is calculated as follows:

$$q_{\text{dyn}} = \frac{C_{\text{in}}V_{\text{in}} - \sum_i C_{\text{out},i} V_i}{M_R} \qquad \text{(S9)}$$

where q_{dyn} is the dynamic adsorption loss (mg/g), C_{in} and $C_{\text{out},i}$ are the injected and i-th effluent nanoparticle concentrations (mg/L), V_{in} is the cumulative injected volume (L), V_i is the volume of the i-th collected fraction (L), and M_R is the dry mass of the core (g).

S6. Porous-Media Transport and Core-Flooding Procedures

S6.1. Core Saturation and Basic Permeability

After cleaning and drying, the core is placed under vacuum conditions for 12 h, and then saturated with baseline brine under vacuum for 12 h. The pore volume is determined by the mass difference before and after saturation, and the porosity is calculated by the ratio of the pore volume to the bulk core volume.

The saturated core is loaded into the core holder and confining pressure is applied. The water-phase permeability is calculated according to Darcy's law:

$$k = \frac{Q\eta L}{A\Delta P} \qquad \text{(S10)}$$

where k is permeability (m^2), Q is volumetric flow rate (m^3/s), η is fluid viscosity (Pa·s), L is core length (m), A is the core cross-sectional area (m^2), and ΔP is the pressure difference across the core (Pa).

S6.2. Nanofluid Transport and Permeability Response

The nanofluid transport system is composed of a constant flow pump, a core holder, a constant temperature chamber, a back pressure regulator, a confining-pressure pump and a differential pressure sensor.

Before injecting nanofluid, the initial permeability was determined by using the base solution at a flow rate of 0.5, 1.0, 2.0 and 4.0 mL/min. Then a predetermined volume of nanofluid was injected, and the pressure difference across the core was continuously recorded. After the nanofluid injection, the core was flushed with the base solution until the pressure difference stabilized to evaluate the irreversible flow resistance changes caused by the retention of nanoparticles.

The permeability retention rate is calculated as follows:

$$R_k = \frac{k_{\text{after}}}{k_{\text{before}}} \times 100\% \qquad \text{(S11)}$$

where k_{before} and k_{after} are the water-phase permeabilities before and after nanofluid treatment, respectively.

The permeability reduction coefficient is calculated as follows:

$$F_k = \frac{k_0}{k_t} \qquad \text{(S12)}$$

where k_0 and k_t are the initial permeability and the permeability at time t, respectively.

The resistance factor and residual resistance factor were calculated as:

$$F_R = \frac{\Delta P_{\text{nf}}}{\Delta P_{\text{w,before}}} \qquad \text{(S13a)}$$

$$F_{RR} = \frac{\Delta P_{\text{w,after}}}{\Delta P_{\text{w,before}}} \qquad \text{(S13b)}$$

where ΔP_{nf} is the pressure difference during nanofluid injection, $\Delta P_{\text{w,before}}$ is the pressure difference during water injection before nanofluid treatment, and $\Delta P_{\text{w,after}}$ is the pressure difference during subsequent water injection.

There is a distinction between static and dynamic permeability-retention tests. In the static test, the permeability is determined after soaking the water-saturated core in 0.30 wt%

nanofluid for 24 hours; in the dynamic test, 1.0 PV nanofluid is injected into the core, and then water flooding is carried out. This distinction is important because the two experiments represent different particle–rock contact and retention processes.

S6.3. Core-Flooding Procedure

The core-flooding procedure was designed with reference to laboratory nanoparticle-assisted oil-displacement protocols reported previously [38,44]. The core flooding experiment was completed on a self-built displacement system, which is composed of a constant-flow pump, a piston-type intermediate container, a high-temperature core holder, a confining-pressure pump, a back-pressure regulator, a differential pressure sensor, a thermostatic chamber and a produced-fluid collection device. The components are connected by pressure-resistant stainless steel pipelines. Before the experiment begins, check the system tightness and rinse all the pipelines with deionised water.

Crude oil is injected into the saturated core at a flow rate of 0.5 mL/min until there is no more water production; then the flow rate is increased to 1.0 mL/min, and injection of crude oil of no less than 3.0 PV is continued to establish a stable initial oil saturation. The oil-saturated core is aged at 60 ° C for 7 days, so that the crude oil components and the sandstone surface reach the adsorption equilibrium again.

Unless otherwise stated, the experimental reference conditions are: nanoparticle concentration 0.30 wt%, pH 8.0, temperature 70 ° C, net confining pressure 1000 psi, back pressure 300 psi, injection flow rate 1.0 mL/min, nanofluid slug 1.0 PV, core permeability approximately 500 mD. The initial water flooding continues until the water cut at the outlet reaches 98% and there is no obvious oil production in a continuous 0.5 PV interval. Then the nanofluid slug of the set volume is injected; if the experimental design includes the soaking step, close both ends of the core for soaking. After soaking, resume water flooding until the water cut at the outlet end reaches 98% again. During the displacement process, the effluent is collected every 0.10 PV, and the oil production, water production and pressure difference across the core are recorded at the same time.

The hydrodynamic particle size range of single-factor experiments is about $50-1000$ nm, the pH range is $2-12$, the concentration of nanoparticles is $0-0.50$ wt%, and the slug size is $0.25-2.0$ PV. In the reservoir adaptability experiment, the temperature is set to 30, 50, 70, 90 and 110 ° C respectively, the net confining pressure is $100-1500$ psi, the injection rate is $0.5-8$ mL/min, the soaking time is $0-50$ h, and the core permeability is about $50-1500$ mD; except for the parameter under investigation, the remaining conditions remain unchanged.

The recovery rate is calculated as follows:

$$R_o = \frac{V_{o,p}}{V_{\mathrm{OOIP}}} \times 100\% \qquad \text{(S14)}$$

where $V_{o,p}$is cumulative oil production and $V_{\mathrm{OOIP}}$is the original oil volume in place.

Incremental recovery after nanofluid injection was calculated as:

$$\Delta R_o = \frac{V_{o,\mathrm{NF}}}{V_{\mathrm{OOIP}}} \times 100\% \qquad \text{(S15)}$$

where $V_{o,\mathrm{NF}}$is the oil produced after nanofluid injection.

Water cut was calculated as:

$$f_w = \frac{V_w}{V_w + V_o} \times 100\% \qquad \text{(S16)}$$

where V_w and V_o are the water and oil volumes collected during the corresponding sampling interval.

Residual oil saturation was calculated as:

$$S_{or} = \frac{V_{\mathrm{OOIP}} - V_{o,p}}{PV} \times 100\% \qquad \text{(S17)}$$

where PV is the core pore volume.

S6.4. Tracer Test and Volumetric Sweep Efficiency

A salt-tracer method was used to evaluate the changes in volumetric sweep efficiency. Before and after nanofluid injection, 0.50 PV of 1.0 wt% KCl tracer solution was injected into the core, and the conductivity of the effluent was continuously monitored.

The average tracer residence time was calculated from the residence-time distribution:

$$\bar{t} = \frac{\int_0^\infty t\,C(t)\,dt}{\int_0^\infty C\,(t)\,dt} \qquad \text{(S18a)}$$

where $C(t)$ is the normalized tracer concentration at time t.

The effective volumetric sweep efficiency was determined as:

$$E_v = \frac{Q\bar{t}}{PV} \times 100\% \qquad \text{(S18b)}$$

where Q is the volumetric flow rate, $\bar{t}$ is the mean residence time, and PV is the pore volume. The above content corresponds to the tracer procedure in Section 2.8 of the manuscript.

S7. Oil–Water Separation, Aging, and Recycling

S7.1. Preparation of Simulated Oily Water

According to the required initial oil concentration, add the predetermined mass of crude oil to the deionised water and stir it mechanically at 1500 rpm at high speed for 30 minutes to obtain a relatively uniform oily water dispersion. The initial oil concentration of the experimental investigation is $100 - 1000$ mg/L.

Simulated oil-containing water should be used within 30 minutes after preparation to reduce the natural separation during the standing process. The particle size of oil droplets is determined by a laser particle size analyzer, and if necessary, it is supplemented by optical microscopy

S7.2. Batch Oil-Removal Experiments

The simulated oil-containing water is made of actual crude oil in the Dagang oil field to retain the representative oil phase composition. However, it should be noted that the water phase is deionised water, not the oil field water collected on the spot.

The predetermined volume of simulated oil-containing water is mixed with nanofluids in a stoppered conical flask, so that the final concentration of nanoparticles is $0.05 - 0.50$ wt%. The sample is shaken at 150 rpm, and the contact time is $5 - 100$ min.

After the treatment, the sample is left for 30 minutes, and then centrifuged at 4000 rpm for 10 min to separate the oil droplets bound with nanoparticles, and then the residual oil concentration in the supernatant is determined.

The initial oil concentration of the experimental investigation is 100 − 1000 mg/L, and the treatment liquid volume is 10 − 100 mL. The temperature-dependent experiment covers 25 − 85 ° C, and the concentration range of Na_2SO_4 is 0 − 5.0 × 10^4 mg/L.

In the ultrasound-assisted experiment, a 40 kHz constant temperature ultrasound bath or a 20 kHz probe ultrasound system is used. The processing time is 0 − 30 min, and the temperature is maintained at 25 ± 2 ° C. Samples corresponding to different ultrasonic times are prepared independently with the same initial volume and temperature to minimise the impact of thermal history effects. The particle size distribution of oil droplets is determined separately before and after ultrasonic treatment.

The oil removal efficiency is calculated as follows:

$$E_{\text{oil}} = \frac{C_0 - C_t}{C_0} \times 100\% \qquad \text{(S19)}$$

where C_0 and C_t are the initial and final oil concentrations in the aqueous phase, respectively. The oil concentration was determined by the n-hexane extraction-UV spectrophotometry described in Section S5.1.

S7.3. Dispersion Stability Index

The dispersion stability of nanofluids is evaluated by the absorbance ratio method. Transfer the freshly prepared sample to the colorimetric tube and store it at 25 °C. At the initial moment and the set ageing time point, collect an equal volume of liquid from the upper third of the sample height, and determine the absorbance at 600 nm.

The stability index is calculated by the following formula:

$$SI_t = \frac{A_t}{A_0} \times 100\% \qquad \text{(S20)}$$

where $A_0$is the initial absorbance and $A_t$is the absorbance after a specified standing or aging time.

A higher SI_tindicates lower particle sedimentation and phase separation. Blank controls are set for oil-containing and oil-free nanofluid systems respectively to minimise the interference caused by oil droplet absorption and nanoparticle light scattering.

S7.4. Long-Term Aging

The long-term ageing experiment is carried out with 0.30 wt% nanofluid. The sample is placed in a sealed brown glass bottle. The liquid volume is about 80% of the bottle capacity, and it is stored at 25 ° C away from light.

The ageing time is set to 0, 1, 3, 7, 14, 21, 30, 45 and 60 days respectively. At each predetermined time point, the sample is gently inverted and mixed without additional ultrasonic treatment, and then its ζ potential, hydrodynamic particle size, stability index, relative viscosity, interfacial tension, oil removal performance and core displacement performance are evaluated.

S7.5. Nanoparticle Recovery and Recycling

After each cycle experiment, the nanoparticles are recovered by centrifugation at 10000 rpm for 15 min. The precipitate is first washed with n-hexane once to remove the adsorbed oil, then twice with anhydrous ethanol and twice with deionised water. The recovered particles are then vacuum-dried at 40 ° C for 12 h.

The recovered nanoparticles are redispersed according to the initial mass concentration, and the dispersion steps are the same as those of fresh nanofluids. A total of five consecutive recycling/reuse cycles are carried out, and the nanoparticle recovery rate and oil removal performance are recorded after each cycle.

If the mass loss of nanoparticles in a cycle exceeds 5%, the subsequent nanofluid will be made according to the actual recovered particle mass, and no fresh particles will be supplemented to make up for the loss.

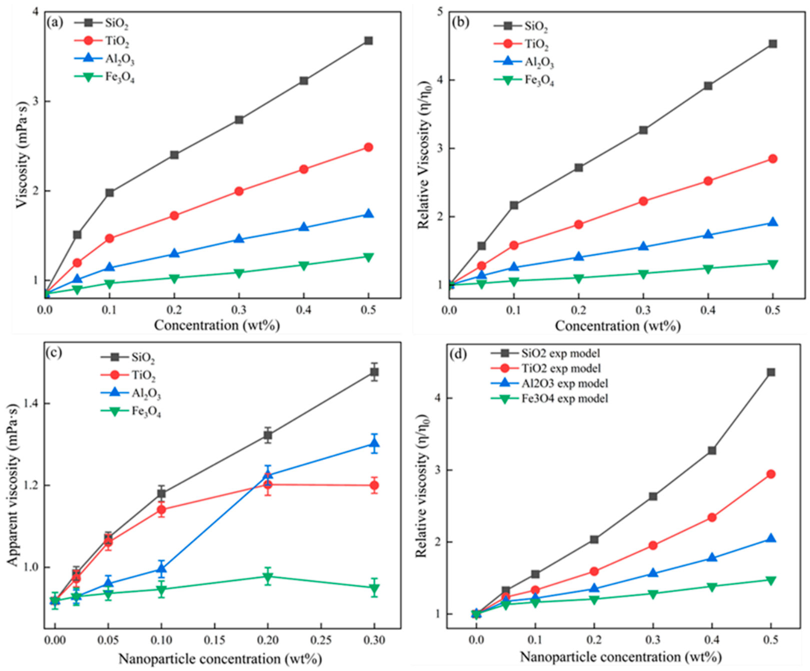

Figure S2. Viscosity changes and empirical model analysis of different metal oxide nanofluids. **(a)** The effect of nanoparticle concentration on dynamic viscosity; **(b)** The effect of nanoparticle concentration on relative viscosity; **(c)** The effect of nanoparticles on apparent viscosity within the low concentration range; **(d)** Changes in relative viscosity of different nanofluids predicted

by empirical models. Error bars represent the standard deviation of the independent replicate measurements.

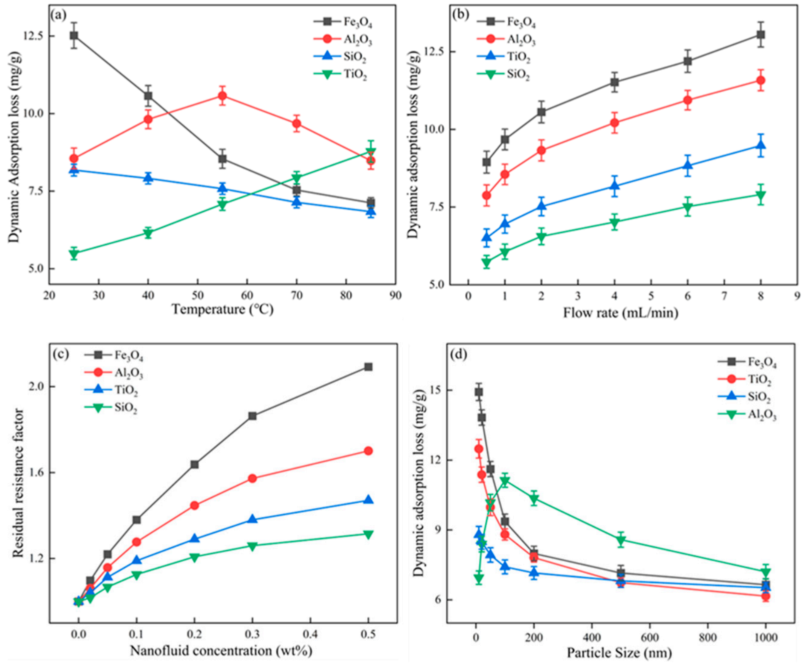

Figure S3. The effects of different factors on the adsorption behavior and flow regulation ability of metal oxide nanofluids. **(a)** The effect of temperature on the dynamic adsorption loss of different nanofluids; **(b)** The effect of flow rate on the dynamic adsorption loss of nanofluids; **(c)** Effect of nanofluid concentration on residual resistance coefficient; **(d)** The effect of particle size on dynamic adsorption loss. Error bars represent the standard deviation of the independent replicate measurements.

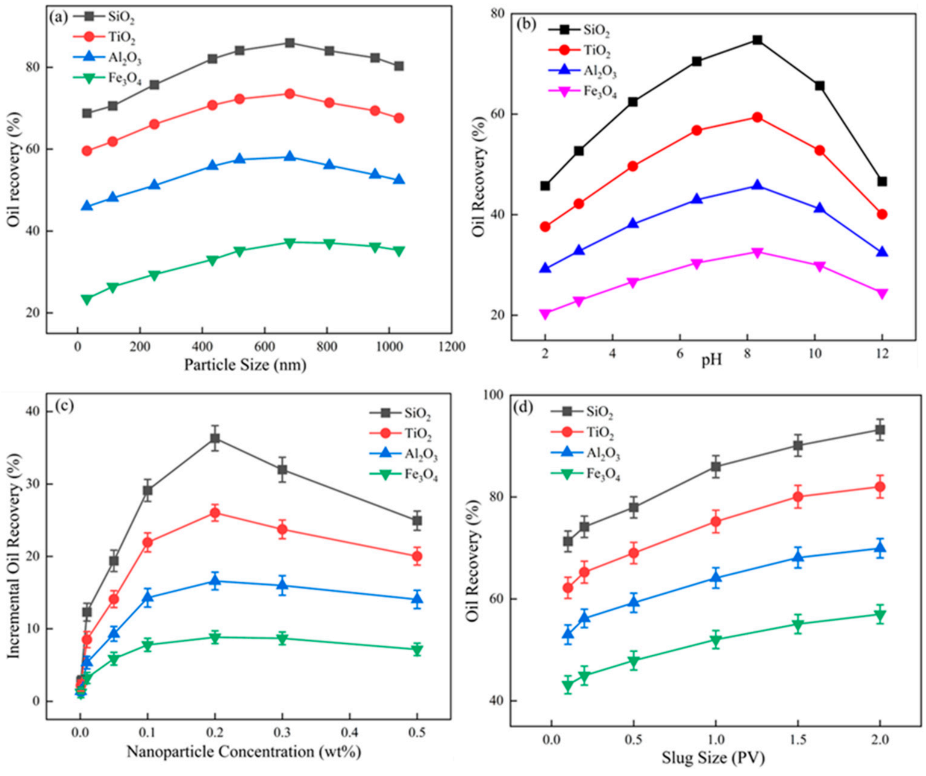

Figure S4. Effects of different experimental parameters on the oil displacement performance of metal oxide nanofluids. **(a)** The effect of different nanoparticle sizes on oil recovery rate; **(b)** Changes in nanofluid oil recovery under different initial pH conditions; **(c)** Effects of different nanoparticle concentrations on incremental oil recovery rates; **(d)** The effect of different injection plug sizes on the final oil recovery rate. Error bars represent the standard deviation of the independent replicate measurements.

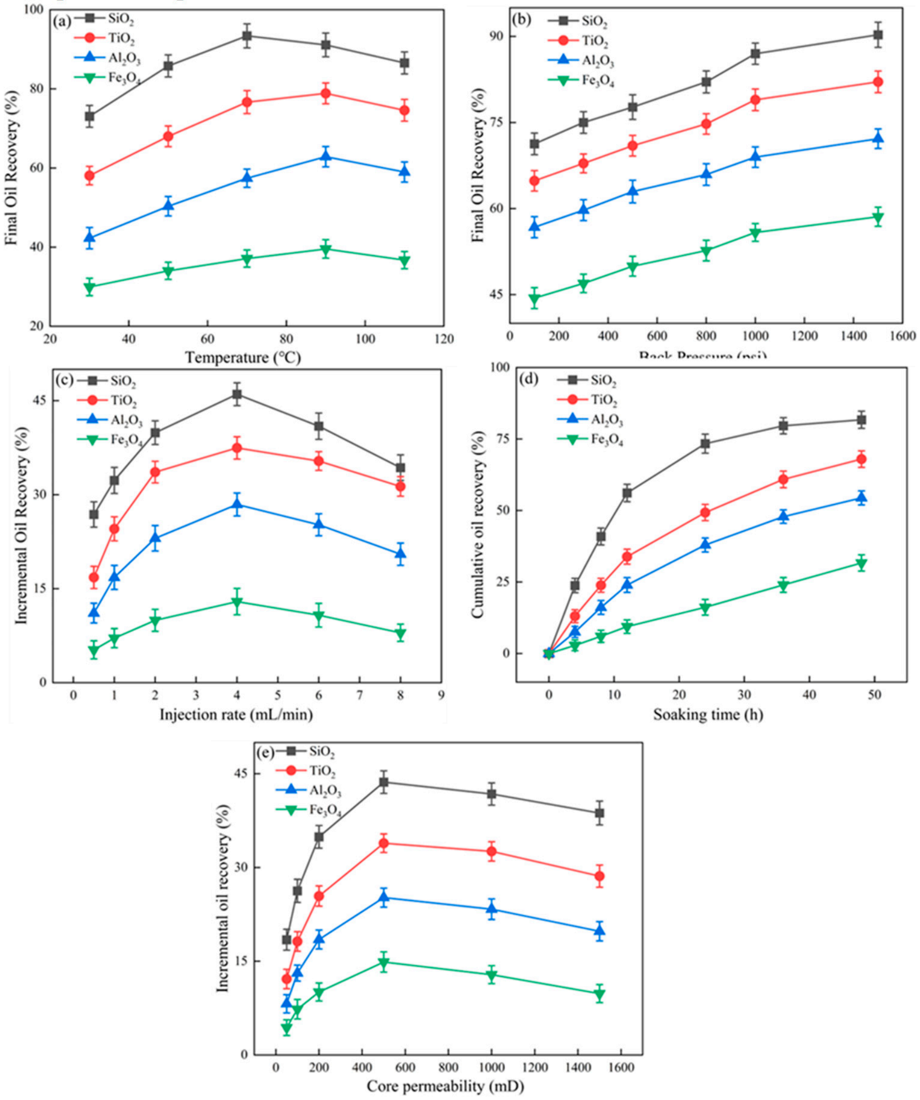

Figure S5. Effects of different reservoir environmental parameters on the oil displacement performance of metal oxide nanofluids. **(a)** The effect of temperature on the final oil recovery rate; **(b)** The impact of confining pressure on the final oil recovery rate; **(c)** The effect of injection flow rate on incremental oil recovery rate; **(d)** The effect of soaking time on cumulative oil recovery rate; (e) The impact of core permeability on incremental oil recovery rate. Error bars represent the standard deviation of the independent replicate measurements.

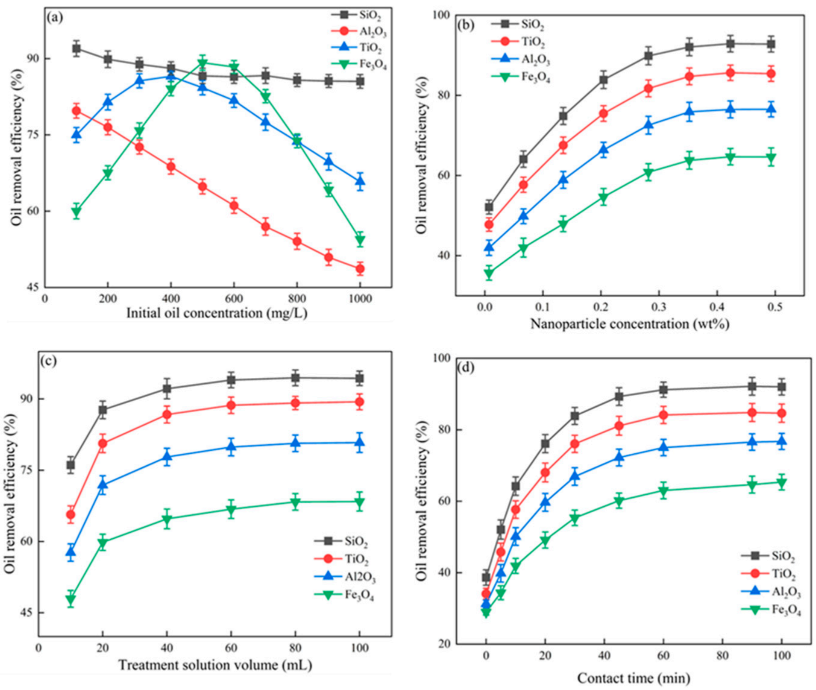

Figure S6. Effects of different experimental conditions on the oil-water separation efficiency of metal oxide nanofluids. **(a)** The effect of initial oil concentration on the oil removal efficiency of nanofluids; **(b)** The effect of nanoparticle concentration on oil removal efficiency; **(c)** The effect of treated liquid volume on oil removal efficiency; **(d)** The effect of contact time on oil removal efficiency. Error bars represent the standard deviation of the independent replicate measurements.

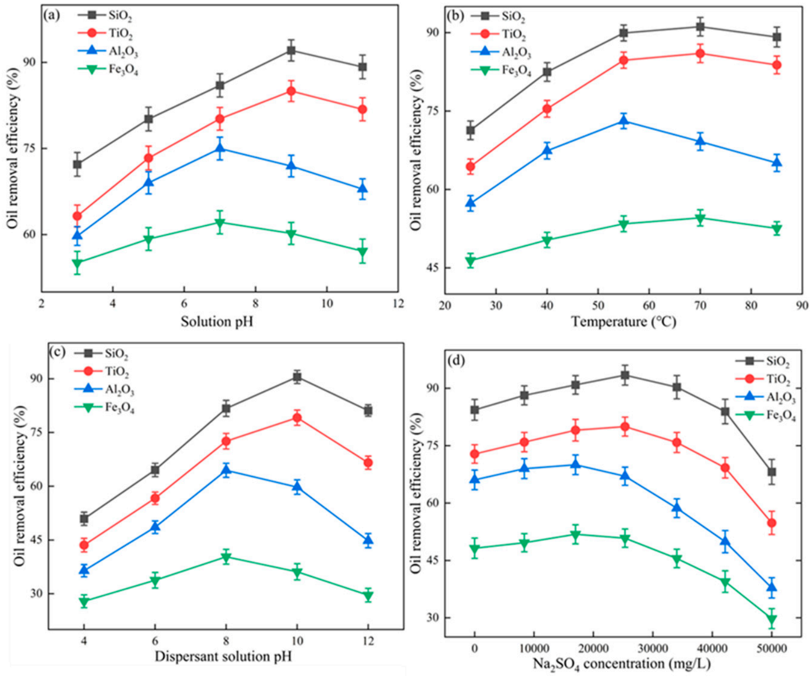

Figure S7. Effects of different operating conditions on the degreasing performance of metal oxide nanofluids. **(a)** Effect of dispersion pH on the oil removal efficiency of nanofluids; **(b)** The effect of temperature on the degreasing efficiency of nanofluids; **(c)** Effect of dispersant solution pH on the degreasing efficiency of nanofluids; **(d)** Effect of sodium sulfate concentration on the

degreasing efficiency of nanofluids. Error bars represent the standard deviation of the independent replicate measurements.

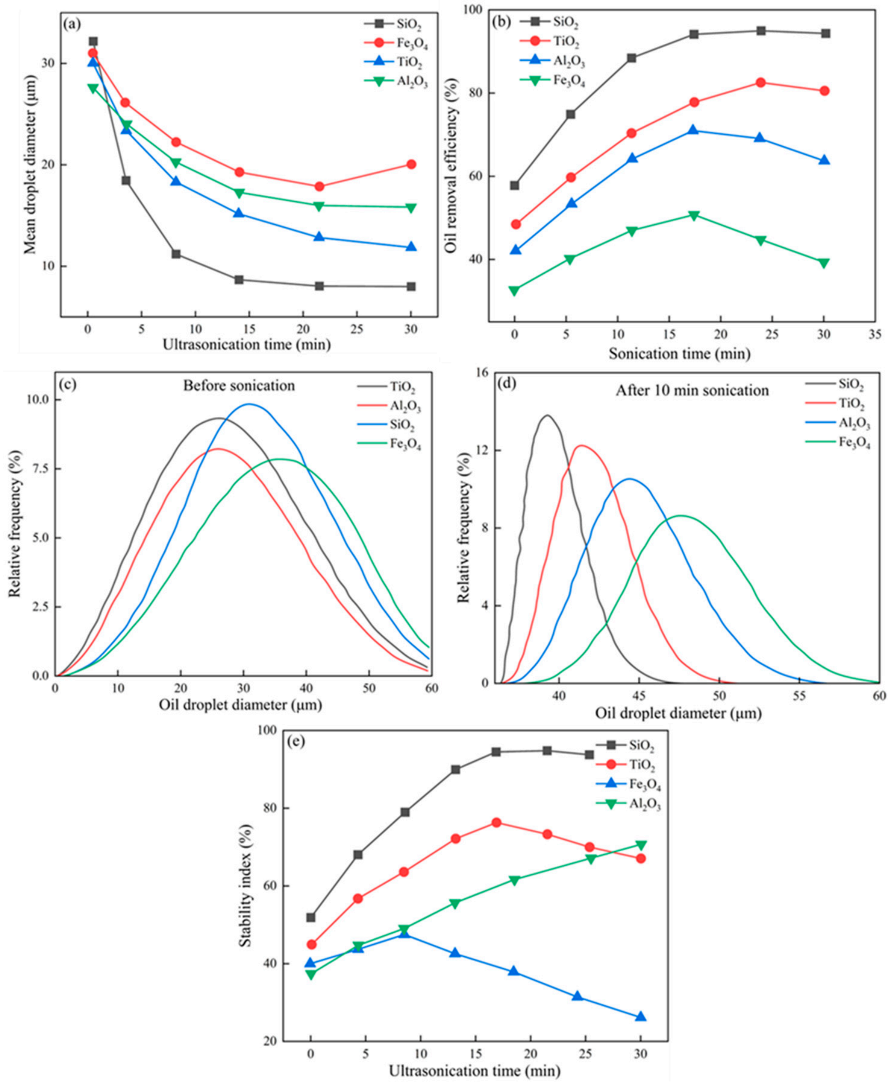

Figure S8. Effect of ultrasonic treatment time on the dispersion behavior and degreasing performance of oil droplets in different metal oxide nanofluids. **(a)** The effect of ultrasound time on the average particle size of oil droplets; **(b)** The effect of ultrasound time on oil removal efficiency; **(c)** Distribution of oil droplet size before ultrasonic treatment; **(d)** Distribution of oil droplet size after 10 minutes of ultrasonic treatment; **(e)** The effect of ultrasound time on the stability index of nanofluids.

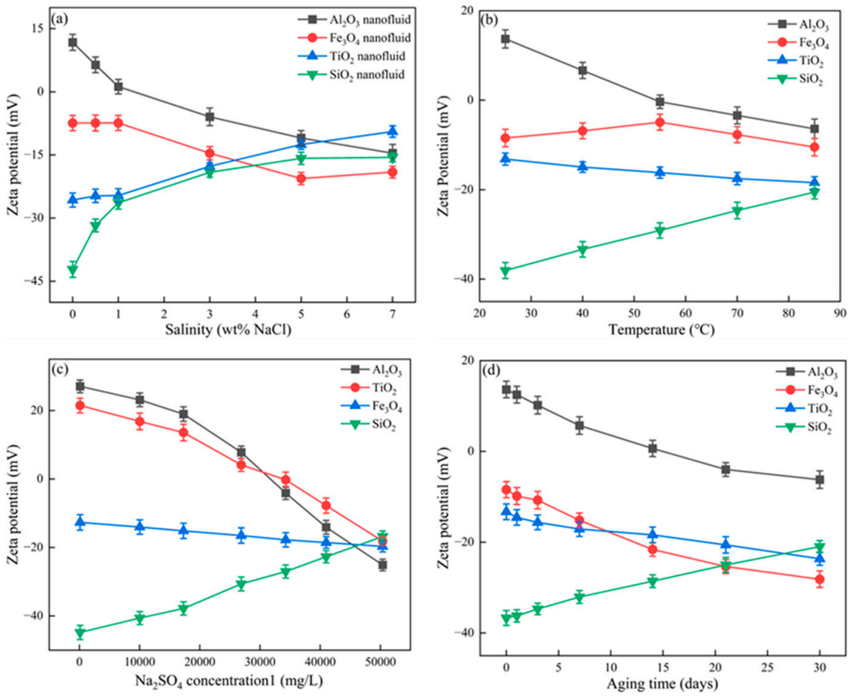

Figure S9. Effects of different environmental factors on the ζ potential of metal oxide nanofluids. **(a)** The effect of NaCl concentration on the ζ potential of nanofluids; **(b)** The effect of temperature on the potential ζ of nanofluids; **(c)** Effect of Na_2SO_4 concentration on the ζ potential of nanofluids; **(d)** The effect of aging time on the ζ potential of nanofluids. Error bars represent the standard deviation of the independent replicate measurements.

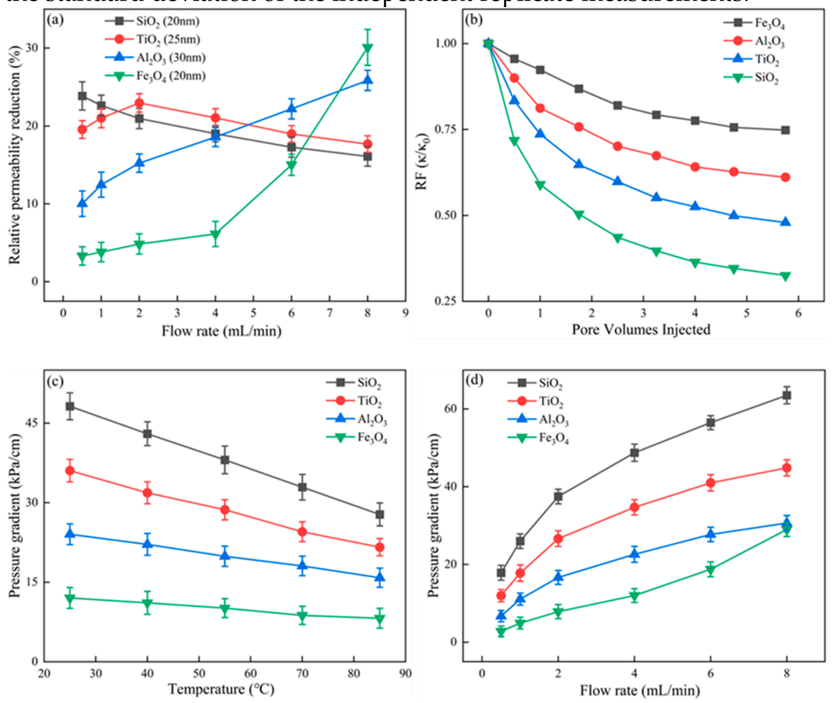

Figure S10. The osmotic flow of different metal oxide nanofluids in porous media varies. **(a)** The rate of relative permeability reduction caused by nanofluids under different flow rate conditions; **(b)** Changes in nanofluid resistance factors under different injected pore volume multiples; **(c)** Changes in the pressure gradient of nanofluids under different temperature conditions; **(d)** Changes in the pressure gradient of nanofluids under different flow rate

conditions. Error bars represent the standard deviation of the independent replicate measurements.
